# Reactive Case Detection and Treatment and Reactive Drug Administration for Reducing Malaria Transmission: A Systematic Review and Meta-Analysis

**DOI:** 10.4269/ajtmh.22-0720

**Published:** 2023-12-20

**Authors:** Laura C. Steinhardt, Achyut KC, Amanda Tiffany, Elizabeth M. Quincer, Leah Loerinc, Nicolas Laramee, Amy Large, Kim A. Lindblade

**Affiliations:** ^1^ Malaria Branch, Division of Parasitic Diseases and Malaria, U.S. Centers for Disease Control and Prevention, Atlanta, Georgia;; ^2^ Global Malaria Programme, World Health Organization, Geneva, Switzerland;; ^3^ Emory School of Medicine, Atlanta, Georgia;; ^4^ Rollins School of Public Health, Emory University, Atlanta, Georgia

## Abstract

Many countries pursuing malaria elimination implement “reactive” strategies targeting household members and neighbors of index cases to reduce transmission. These strategies include reactive case detection and treatment (RACDT; testing and treating those positive) and reactive drug administration (RDA; providing antimalarials without testing). We conducted systematic reviews of RACDT and RDA to assess their effect on reducing malaria transmission and gathered evidence about key contextual factors important to their implementation. Two reviewers screened titles/abstracts and full-text records using defined criteria (Patient = those in malaria-endemic/receptive areas; Intervention = RACDT or RDA; Comparison = standard of care; Outcome = malaria incidence/prevalence) and abstracted data for meta-analyses. The Grading of Recommendations, Assessment, Development, and Evaluations approach was used to rate certainty of evidence (CoE) for each outcome. Of 1,460 records screened, reviewers identified five RACDT studies (three cluster-randomized controlled trials [cRCTs] and two nonrandomized studies [NRS]) and seven RDA studies (six cRCTs and one NRS); three cRCTs comparing RDA to RACDT were included in both reviews. Compared with RDA, RACDT was associated with nonsignificantly higher parasite prevalence (odds ratio [OR] = 1.85; 95% CI: 0.96–3.57; one study) and malaria incidence (rate ratio [RR] = 1.30; 95% CI: 0.94–1.79; three studies), both very low CoE. Compared with control or RACDT, RDA was associated with non-significantly lower parasite incidence (RR = 0.73; 95% CI: 0.36–1.47; 2 studies, moderate CoE), prevalence (OR = 0.78; 95% CI: 0.52–1.17; 4 studies, low CoE), and malaria incidence (RR = 0.93; 95% CI: 0.82–1.05; six studies, moderate CoE). Evidence for reactive strategies’ impact on malaria transmission is limited, especially for RACDT, but suggests RDA might be more effective.

## INTRODUCTION

Once malaria transmission declines to very low levels, case-based surveillance with case investigations to determine the likely location of infection becomes feasible. Given the clustered nature of malaria cases at very low transmission levels,^1^^,^^2^ many countries pursuing malaria elimination have implemented interventions around the likely location of infection of people with confirmed malaria to strengthen surveillance and further reduce or interrupt transmission. Referred to as “reactive” strategies because they are initiated in response to a person with malaria (index case), these actions target those who might have been exposed at the same time, including household members and, in some cases, neighbors. If reactive strategies are effective at targeting a large proportion of the reservoir of infection, or at preventing infections in the highest-risk populations, there should be an overall reduction in transmission of malaria. Two reactive strategies that have been deployed by countries that have eliminated malaria are reactive case detection and treatment (RACDT) and reactive drug administration (RDA).

RACDT is a form of active case detection that has become increasingly common in very low transmission settings in the past decade and involves parasitological testing and treatment of those found positive around an index case. As of 2014, 13 of 14 countries in the Asia Pacific Malaria Elimination Network and several countries in Africa were implementing some form of RACDT, either as a widespread programmatic intervention or as a pilot.^3^ Operational approaches to RACDT vary widely, with some RACDT programs or studies testing only members of the index household,^4^ whereas others include all residents living within a certain radius of the index household^5^^–^^7^; some RACDT programs also attempt to test individuals exposed to infection at the same time as the index case, such as cotravelers or coworkers.^8^ Previous studies have found that proximity to the index case,^9^^,^^10^ a more sensitive diagnostic method,^3^^,^^10^^,^^11^ timeliness of the response,^9^ history of fever,^12^ and recent travel^11^^,^^12^ were positively related to the likelihood of finding secondary cases.

As a surveillance strategy, RACDT improves the sensitivity of the surveillance system in very low transmission settings by increasing testing in the areas where additional cases are most likely to be found. However, RACDT is often undertaken with the goal of reducing malaria transmission by identifying and treating clinical infections more quickly or by detecting and treating afebrile infections that would otherwise not seek care. Despite the popularity of RACDT, evidence of its effectiveness in reducing malaria transmission is limited, and a 2016 systematic review concluded that it was labor-intensive and the benefits were not clear.^13^

RDA is a form of chemoprevention, similar to mass drug administration, that treats all existing infections and prevents new infections over the prophylactic period of the drug. RDA targets the same population as RACDT, that is, those living with or near an index case along with cotravelers and coworkers. However, RDA overcomes two limitations of RACDT: RDA clears low-density infections that might be missed by current point-of-care diagnostics, and everyone, not just those infected, is protected from infection during the prophylactic period.^14^ Modeling work supports the hypothesis that RDA will avert more cases than RACDT.^15^

The two strategies, RACDT and RDA, not only differ in their approach to reducing transmission but are likely to pose different logistical and operational challenges, be perceived differently by communities and malaria program staff, and affect health equity in dissimilar ways. For example, parasitological testing of individuals who do not feel ill may confuse participants, and blood collection, even from a finger-prick, can raise various concerns.^16^^,^^17^ However, for RACDT, trust in the results of malaria rapid diagnostic tests (RDTs) can improve acceptance of, and adherence to, antimalarial medicine.^18^ Conversely, in RDA, provision of a full-course of antimalarials to individuals who have not been tested and who do not feel ill may raise concern over the side effects of the medicine, while provision of medicine to someone who is not infected raises ethical and equity issues.^19^^,^^20^ The logistical challenge of determining who is eligible for antimalarial medicine in an RDA program is likely to be very different from implementing parasitological testing for the whole population. The determination of which strategy should be recommended depends on these and similar factors as much as it does on the effectiveness of the approach in reducing malaria transmission.

Despite growing interest in deploying reactive strategies in very low transmission settings, and even in higher transmission settings, there has not been a recent synthesis of their effectiveness. To inform development of WHO guidelines on reactive strategies in areas approaching elimination, we conducted a systematic literature review on the effectiveness of RACDT and RDA and summarized evidence about key contextual factors important to their implementation.

## METHODS

The methods for this systematic review have been described extensively elsewhere^21^ (see the Methods section of the supplemental materials) and in the prospectively published protocol [PROSPERO registration numbers CRD42021249329 and CRD4202124973]. Search terms used are presented in Supplemental Table 1. An initial search was run through November 2020 and later updated through March 2022. Specific attributes of the methods for this review follow.

### Population, intervention, comparator, and outcomes.

The key questions for the RACDT and RDA reviews were the following: Should people residing with or near a confirmed malaria case be tested for malaria at approximately the same time and treated if positive to reduce human malaria transmission? Should people residing with or near a confirmed malaria case be given a full therapeutic course of an antimalarial medication at approximately the same time to reduce human malaria transmission? RACDT was defined as testing and treating individuals residing with or near a confirmed malaria case within 1 month of index case diagnosis. Individuals with parasitologically confirmed malaria infections were treated as per the national treatment guidelines. Parasitological confirmation of malaria infection in the RACDT strategy could include RDT, microscopy, or molecular methods such as loop-mediated isothermal amplification (LAMP) and polymerase chain reaction (PCR). In some cases, RACDT could include screening for symptoms (e.g., fever) before testing. RDA was defined as providing a full therapeutic course of an antimalarial medicine to people residing with or near a confirmed malaria case within 1 month of index case diagnosis. The main comparator for both interventions was no intervention (e.g., no RDA or no RACDT). However, because of the limited number of studies, we also included reports where RDA and RACDT were compared with another intervention.

Beneficial outcomes included community-level malaria transmission and prevalence of malaria among those receiving the intervention, as described in the supplemental materials, Methods section. The primary harmful outcome was adverse drug events among participants. Contextual factors (see supplemental materials) were also abstracted and summarized.

### Risk of bias (quality) assessment.

Two members of the review team independently assessed the risk of bias for each included study and for each specific outcome. For cluster-randomized controlled trials (cRCTs), the revised Cochrane Risk of Bias (ROB 2) tool^22^ was used, along with other considerations in Chapter 8 of the Cochrane Handbook, *Assessing Risk of Bias in a Randomized Trial*^23^ to rate each cRCT as low risk of bias, some concerns, or high risk of bias. We assessed nonrandomized studies (NRS) for risk of bias using slightly modified criteria from Table 2 in a publication on Grading of Recommendations, Assessment, Development, and Evaluations (GRADE) and risk of bias in NRS.^24^

### Data synthesis.

For study designs with a control group, the effect of the intervention was compared using risk ratios or odds ratios (OR) for dichotomous outcomes and rate ratios (RR) for rates or counts, for example, using Poisson regression or negative binomial analyses. For cRCTs, effect measures adjusted for clustering and for other relevant covariates, as specified in the trial protocol and reported by study authors, constituted the primary measure of treatment effect. When possible, we performed sensitivity analyses using unadjusted (for other covariates) effect measures to investigate the robustness of our analyses. We attempted to contact study authors to obtain any missing data. No data imputation was applied.

For outcomes with more than one study contributing data, we assessed heterogeneity of main effects by examining forest plots for overlapping CIs. Statistical heterogeneity was examined using the *I*^2^ statistic. If there was either considerable heterogeneity (*I*^2^ statistic value between 75% and 100%) or inconsistency in the direction of the effect, or both, then we did not perform a meta-analysis. We used fixed effects meta-analysis to combine data if heterogeneity was absent (*I*^2^ statistic <50%), when there were only two studies, or when there were data from multiple small but biased studies and one well-conducted study. Otherwise, we combined data using a random effects meta-analysis and reported a pooled treatment effect. Potential effect modifiers (Supplemental Table 2) were prespecified for subgroup analyses, but there were insufficient data to permit any subgroup analyses. NRS were included in separate meta-analyses. Meta-analysis was carried out using Review Manager software, version 5.4.1 (The Cochrane Collaboration, London, UK). Information on contextual factors was presented in narrative summaries.

### Grading the certainty of the evidence.

The certainty of evidence was evaluated using the GRADE approach,^25^ and each outcome was rated separately for evidence contributed by the randomized and nonrandomized studies as described by Balshem et al.,^26^ using the GRADEPro software. Additional details can be found in the methods section of the supplemental materials.

## RESULTS

**Figure 1.**
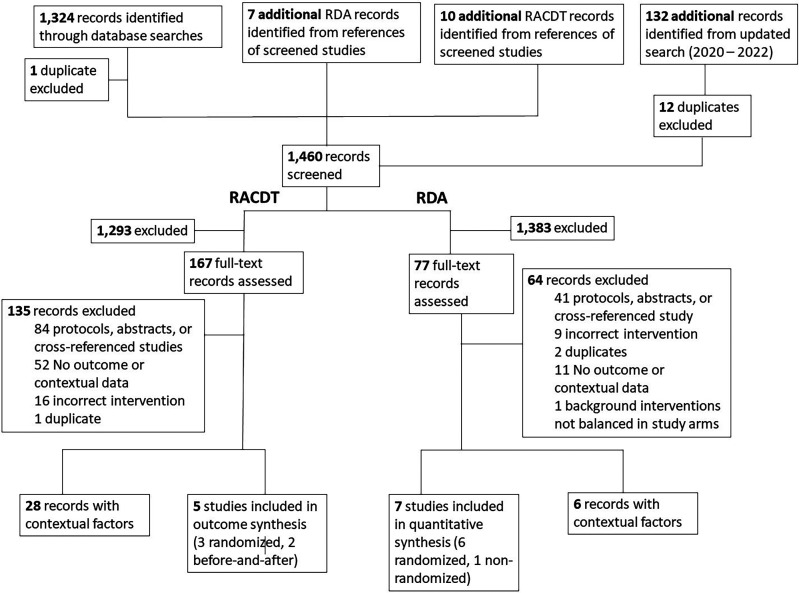
PRISMA diagram. RDA = reactive drug administration; RACDT = reactive case detection and treatment.

A total of 1,323 unique records were identified (after one duplicate removal) from the initial database searches; an additional 120 articles were added (after 12 duplicates removals) from the updated search through March 2022 (Figure 1). Ten additional articles were identified for RACDT and seven for RDA during full text screening, yielding a total of 1,460 records screened. We conducted full-text reviews of 167 studies of RACDT and 77 of RDA. After full text screening, we identified five studies with outcome data for RACDT (three randomized and two nonrandomized) and seven for RDA (six randomized and one nonrandomized; reasons for exclusion are listed in Figure 1). Twenty-eight articles had information on contextual factors for RACDT and six for RDA.

Three cRCTs in sub-Saharan Africa compared RDA to RACDT (Table 1 and Supplemental Table 3) because the latter was considered standard of care. These three cRCTs were the only randomized studies of RACDT, and therefore we included them in the RACDT review by taking the inverse of the RDA to RACDT comparison. Three additional cRCTs compared RDA with no RDA. Two NRS, one in Zambia (Searle 2020^27^) and one in Brazil (Fontoura 2016^28^), included outcome data for RACDT, and one NRS in Peru (Quispe 2018)^29^ assessed the impact of RDA. Of the nine studies included, seven were from sub-Saharan Africa with nearly all infections due to *Plasmodium falciparum*, and two of the three NRS were from Latin America with predominantly *P. vivax* parasites. All studies except one (Eisele 2020—HIGH^30^) were from low-transmission settings; the Eisele 2020—HIGH^30^ study took place in higher transmission strata (*P. falciparum* prevalence of ≥10%) in the Zambia cluster-randomized trial; the other half of this trial that took place in low-transmission (<10% *P. falciparum* prevalence areas; Eisele 2020—LOW^30^ was considered as a separate study).

**Table 1:** Selected characteristics of studies included for outcome data

Study	Country	Year(s) of Study	Study Design	Study Arms	Index Case Detection	Malaria Transmission	RACD Diagnostic	Drug Used for RACDT	Drug Used for RDA	Radius for RACDT	Radius for RDA
Randomized											
RACDT and RDA											
Bridges 2021^31^	Zambia	2016–18	cRCT	Two-arm trial comparing RDA with RACD	PS	12.7 per 1,000 API in 2015	RDT (HRP2)	AL	DP	140 m	140 m
Hsiang 2020^32^	Namibia	2017	cRCT	Four-arm trial with factorial design: RDA, reactive IRS (+RACD), RDA+reactive IRS, and RACD)	PS	35.9 per 1,000 API in 2016	RDT (HRP2/pLDH) (*Pf*/PAN)	AL+low dose PQ	AL	500 m	500 m
Vilakati 2021^33^	Eswatini	2016–17	cRCT	Two-arm trial comparing RDA with RACD	PS	6.3 per 1,000 API in RDA arm, 2012–2015	RDT (HRP2)	AL	DP	500 m	200 m
RDA only											
Eisele 2020–LOW^30^	Zambia	2015–16	cRCT	Three-arm trial with two intervention arms (MDA and RDA) and one control arm	AS (four rounds)	<10% *Pf*PR among CU5	–	–	DP	HH only	HH only
Eisele 2020–HIGH^30^	Zambia	2015–16	cRCT	Three-arm trial with two intervention arms (MDA and RDA) and one control arm	AS (four rounds)	≥10% PfPR among CU5	–	–	DP	HH only	HH only
Okebe 2021^34^	The Gambia	2017–18	cRCT	Two-arm trial comparing RDA with control*	PS	Malaria prevalence in 2012 in two study areas was 4.6% and 8.4% by PCR	–	–	DP	Compound only (if febrile)	Compound only
Nonrandomized											
RACDT only											
Searle 2020^27^	Zambia	2016–18	Uncontrolled before–after	RACD only	PS	1.3% *Pf*PR in 2013	RDT (HRP2)	AL	–	250 m	–
Fontoura 2016^28^	Amazonia (Brazil)	2013	Controlled before–after	RACD in index/neighbor HH, no RACD in control HH	PS	Five per 1,000 in 2012	Thick smear microscopy	CQ+low- dose PQ	–	Five nearest HH	–
RDA only											
Quispe 2018^29^	Peru	2009–10	NRS (quasi- experimental with control)	Comparison of districts with RDA vs. districts with no RDA	PS	8.2 per 1,000 API in 2010	–	–	CQ+PQ	HH members + social contacts	–

AL = artemether-lumefantrine; API = annual parasite incidence; AS = active surveillance; CQ = chloroquine; cRCT = cluster randomized-controlled trial; CU5 = Children <5 years of age; DP = dihyroartemisinin-piperaquine; HH = households; HRP2 = histidine-rich protein 2; IRS = indoor residual spraying; MDA = mass drug administration; NRS = nonrandomized study; PCR = polymerase chain reaction; *Pf* = *Plasmodium falciparum*; *Pf*PR = *Plasmodium falciparum* parasite rate; pLDH = *Plasmodium* lactate dehydrogenase PQ = primaquine; PS = passive surveillance;* Pv* = *Plasmodium vivax*; RACDT = reactive case detection; RDA = reactive drug administration; RDT = rapid diagnostic test.

In all studies except two (Eisele 2020^30^—LOW and Eisele 2020—HIGH^30^), RDA was conducted in response to a passively detected case through the routine health system. In the Eisele 2020 trial, all cases found during four rounds of active surveillance triggered an RDA response.

In the studies from sub-Saharan Africa, the area targeted around the index case ranged from index household members only (Eisele 2020—HIGH and Eisele 2020—LOW^30^) to all residents of households within 500 m of the index case (Hsiang 2020^6^). All studies in sub-Saharan Africa used standard doses of artemether-lumefantrine (AL) for RACDT and dihydroartemisinin-piperaquine (DP) for RDA except one in Namibia, which used AL plus low-dose primaquine for RACDT and AL for RDA. A nonrandomized study from Peru (Quispe 2018^29^) used chloroquine (25 mg/kg for 72 hours) plus primaquine (0.5 mg/kg for 7 days) for treating household members (excluding children <5 years, elderly members >65 years, pregnant women) and social contacts of index cases. Only one trial in The Gambia (Okebe 2021^34^) screened compound members for symptoms (fever) before RACDT.

Malaria infection prevalence was typically measured by cross-sectional surveys in the study area, using either RDTs or PCR (Table 2). Clinical malaria incidence was measured in included studies using routine data on confirmed malaria cases (either microscopy or RDT) from the health system facilities (and in some cases, community health workers) in the study area. The two studies with infection incidence as an outcome used an 18-month cohort of individuals who had monthly finger prick blood collections for PCR (Table 2).

**Table 2:** Details of outcome measurement in included studies

					Infection Prevalence*			Clinical Malaria Incidence		
Study	Country	Year(s) of Study	Study Design	Study Arms	Outcome Population	Timepoint(s) Outcome Measured	Outcome Diagnostic	Outcome Population	Timepoint(s) Outcome Measured	Outcome Diagnostic
Randomized										
RACD and RDA										
Bridges 2021^31^	Zambia	2016–18	cRCT	Two-arm trial comparing RDA with RACD	Children <15 years through x-sectional survey	May 2018	PCR, RDT	Patients at 16 health facilities in study area	May 2016–May 2018	RDT (from DHIS2 data)
Hsiang 2020^32^	Namibia	2017	cRCT	Four-arm trial with factorial design: RDA, reactive IRS (+RACD), RDA+reactive IRS, and RACD)	All ages in a cross- sectional survey	End of malaria season	PCR	Patients at 11 health facilities in study area	Starting 8 weeks after first intervention in cluster	RDT or microscopy
Vilakati 2021^33^	Eswatini	2016–17	cRCT	Two-arm trial comparing RDA with RACD	–	–	–	Patients at facilities in study area	Starting with first passively detected case in each cluster	Unspecified
RDA only										
Eisele 2020– LOW^30^	Zambia	2015–16	cRCT	Three-arm trial with two intervention arms (MDA and RDA) and one control arm	Children <6 years in two cross-sectio-l surveys	April/May 2014 and April/May 2015	RDT	Patients visiting CHWs and 20 health facilities	Duration of intervention	RDT or microscopy
Eisele 2020– HIGH^30^	Zambia	2015–16	cRCT	Three-arm trial with two intervention arms (MDA and RDA) and one control arm	Children <6 years in two cross-sectional surveys	April/May 2014 and April/May 2015	RDT	Patients visiting CHWs and 20 health facilities	Duration of intervention	RDT or microscopy
Okebe 2021^34^	The Gambia	2017–18	cRCT	Two-arm trial comparing RDA with control*	Participants of all ages in two cross-sectional surveys	2017 and 2018	RDT	Patients visiting health facilities and VHWs	Duration of intervention	RDT
Nonrandomized										
RACD only										
Searle 2020^27^	Zambia	2016-18	Uncontrolled before-after	RACD only	RACD households	Days 0, 30, and 90 after RACD	PCR	–	–	–
Fontoura 2016^28^	Amazonia (Brazil)	2013	Controlled before-after	RACD in index/ neighbor households, no RACD in control households	RACD and control households	Days 0, 30, 60, and 180 after RACD	PCR	–	–	–
RDA only										
Quispe 2018^29^	Peru	2009–10	NRS (quasi- experimental with control)	Comparison of districts with RDA vs. districts with no RDA	–	–	–	Patients in the health system in study area	Duration of intervention	Unspecified

CHW = community health worker; cRCT = cluster randomized-controlled trial; DHIS2 = District Health Information Software 2; MDA = mass drug administration; NRS = nonrandomized study; PCR = polymerase chain reaction; *Pf* = *Plasmodium falciparum; Pv* = *Plasmodium vivax*; RACD = reactive case detection; RDA = reactive drug administration; RDT = rapid diagnostic test; VHW = village health worker.

* The only studies measuring infection incidence were Eisele 2020 HIGH and Eisele 2020 LOW; these studies used a cohort of participants aged 3 months or older over 18 months (January 2015 to May 2016) and monthly PCR for infection detection.

### Risk of bias in included studies.

Risk of bias was generally low across all aspects of the randomized trials except for the outcome of adverse events, for which a much stronger focus on adverse events in the RDA arm compared with the RACDT or comparison arm led to ratings of high risk of bias. The controlled before-and-after RACDT study from Brazil (Fontoura 2016^28^) was rated as low risk across all domains, whereas the uncontrolled before-and-after study from Zambia (Searle 2020^27^) was rated as high risk of bias with some concerns on two domains. The Quispe 2018^29^ study, the one nonrandomized RDA study, was rated as ‘Some concerns’ for four of five risk of bias domains and thus was judged to be at high risk of bias overall.

Assessments for risk of bias for each study are summarized in Supplemental Figures 1–7, with additional details behind these judgments provided in Supplemental Table 3.

### Impact of RACDT and RDA.

#### Incidence of malaria infection.

**Figure 2.**
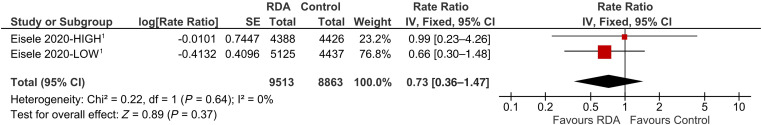
Forest plot of comparison: reactive drug administration (RDA) versus no RDA on incidence of malaria infection. ^1^Negative binomial regression with random effect (cluster level) and adjusted for first month of incidence, age, gender, household socioeconomic class, vector control, rainfall, enhanced vegetation index, and elevation.

No RACDT studies reported on reductions in the incidence of malaria infection. Two studies of RDA (Eisele 2020—HIGH and Eisele 2020—LOW^30^) in Zambia provided data on the incidence of malaria infection based on follow-up of cohorts of individuals in both arms with infection data derived from PCR using dried blood spots. Neither the high-transmission nor the low-transmission study indicated a significant reduction in incidence of malaria infection. The pooled estimate of the two trials indicated a nonsignificant reduction in incidence of infection after RDA (RR: 0.73; 95% CI: 0.36–1.47) (Figure 2).

#### Prevalence of malaria infection.

**Figure 3.**
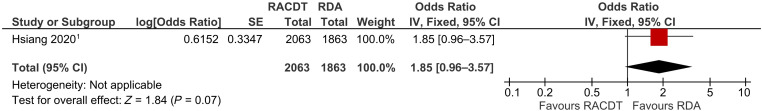
Forest plot of comparison: reactive case detection and treatment (RACDT) versus reactive drug administration (RDA) on prevalence of malaria infection. ^1^The 95% CI upper limit presented here is artificially lower than in the published paper (odds ratio = 1.85, 95% CI: 0.96–20.00), because the authors of the Namibia trial calculated the effect size using marginal effects post-estimation (to account for reactive indoor residual spraying [IRS] in half the clusters) after a regression model, and Review Manager software can only accommodate balanced CIs. Effect size from (nonlinear) marginal effect post-estimation from generalized estimating equations (GEE) model using a logit function adjusted for reactive IRS, the interaction between reactive IRS and RDA, 2016 incidence of local cases, index case level and target population coverage for RDA or RACDT, response time, and co-interventions by Namibia Ministry of Health. Unadjusted effect size (from postestimation marginal effect of RDA from GEE model using a logit function adjusted for reactive IRS, the interaction between reactive IRS and RDA but no other covariates): 0.95 (95% CI: 0.48–33.3).

**Figure 4.**
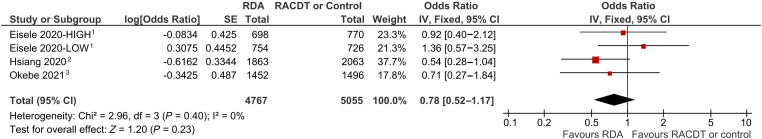
Forest plot of comparison: reactive drug administration (RDA) versus no RDA/reactive case detection and treatment (RACDT) on prevalence of malaria infection. ^1^Random effects logistic regression model adjusted for child age (in years), gender, household wealth from an asset index, rainfall, enhanced vegetation index, household elevation, and household protection by long-lasting insecticidal nets and indoor residual spraying (IRS). ^2^The 95% Cl lower limit is higher here than in the published paper (odds ratio = 0.54, 95% CI: 0.05–1.04) because the authors of the Namibia trial calculated the effect size using marginal effects post-estimation (to account for reactive IRS in half the clusters) after a regression model, and Review Manager software can only accommodate balanced CIs. Effect size from (nonlinear) marginal effect post-estimation from generalized estimating equations (GEE) model using a logit function with variables for RDA, reactive IRS, the interaction between reactive IRS and RDA, and adjusted for 2016 incidence of local cases. Unadjusted effect size (from post-estimation marginal effect of RDA from GEE model using a logit function with variables for RDA, reactive IRS, the interaction between reactive IRS and RDA but no other covariates): 1.05 (0.03–2.07). ^3^Random effects logistic regression (random effect for health facility) adjusted for age. Unadjusted odds ratio: 0.73 (95% CI: 0.27–1.94).

One study in Namibia (Hsiang 2020^32^) compared parasite prevalence between RDA and RACDT arms using post-intervention cross-sectional surveys; the inverse comparison showed a higher odds of malaria infection in the RACDT arm compared with RDA, although the confidence interval included one (OR: 1.85; 95% CI: 0.96–3.57) (Figure 3). The Namibia study and three others contributed to a pooled estimate of RDA versus no RDA or RACDT that showed a nonsignificant reduction of parasite prevalence (pooled OR: 0.78; 95% CI: 0.52–1.17) (Figure 4). A sensitivity analysis omitting the Eisele studies, given their different approach using four rounds of active case detection as opposed to continuous passive case detection, showed a marginally significant reduction in parasite prevalence (pooled OR: 0.59; 95% CI: 0.34–1.01) (Supplemental Figure 8).

#### Incidence of clinical malaria.

**Figure 5.**
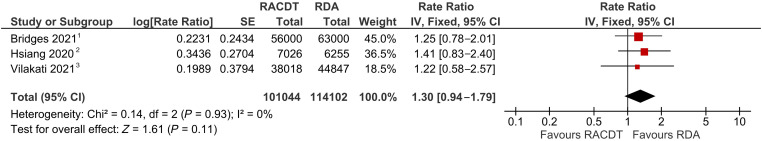
Forest plot of comparison: reactive case detection and treatment (RACDT) versus reactive drug administration (RDA) on clinical malaria incidence. ^1^Negative binomial analysis of monthly facility cases (random intercept for facility); adjusted for previous month’s cases, normalized difference vegetation index, precipitation, altitude, nighttime light, number of RDTs done each month, and seasonality (Fourier term). Unadjusted estimate: 0.93 (0.77–2.00). ^2^The 95% Cl upper limit is lower here than in the published paper (1.41, 95% CI: 0.83–4.55) because the authors of the Namibia trial calculated the effect size using marginal effects post-estimation (to account for reactive indoor residual spraying [IRS] in half the clusters) after a regression model, and Review Manager software can only accommodate balanced CIs. Estimate from (nonlinear) marginal effect post-estimation from a negative binomial model with offset for cluster-level person time; adjusted for reactive vector control, interaction between RDA and reactive vector control, 2016 incidence of local cases, index case level and target population coverage for RACDT or RDA, response time, and cointerventions by Namibia Ministry of Health. Unadjusted marginal effects from post-estimation (from unadjusted negative binomial model with terms for RACDT, reactive IRS, and the interaction between the two, with offset for cluster-level person time): 1.22 (0.73–3.85). ^3^Negative binomial regression model of local cases with offset for person-time and adjusted for baseline (2014–2015) incidence of local cases. Unadjusted estimate: 0.94 (95% CI: 0.51–1.75).

Three randomized trials (Bridges 2021,^35^ Hsiang 2020,^32^ and Vilakati 2021^33^) compared the effect of RDA to RACDT on the incidence of clinical malaria using health facility data. We used the inverse of the outcome measure to assess the impact of RACDT (versus RDA). Two trials, (Bridges 2021^31^ in Zambia and Vilakati 2021^33^ in Eswatini), found (nonsignificantly) higher clinical malaria incidence in the RACDT arms compared with the RDA arms (Zambia RR: 1.25; 95% CI: 0.78–2.01; Eswatini RR 1.22; 95% CI: 0.58–2.57). A third trial in Namibia (Hsiang 2020^32^) reported a slightly larger but still nonsignificant increase in clinical malaria incidence in the RACDT arm compared with the RDA arm (RR: 1.41; 95% CI: 0.83–4.55). The pooled estimate across the three trials comparing RACDT to RDA showed a nonsignificant increase in clinical malaria incidence (pooled RR: 1.30; 95% CI: 0.94–1.79) (Figure 5).

**Figure 6.**
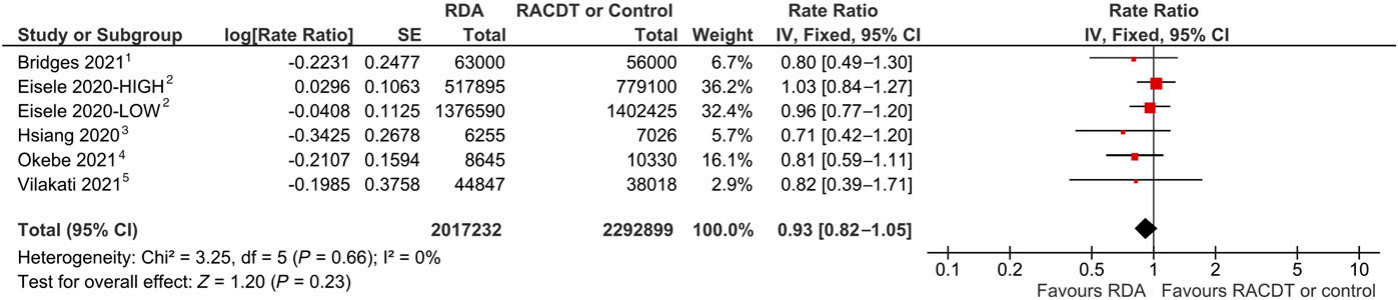
Forest plot of comparison: reactive drug administration (RDA) versus no RDA/reactive case detection and treatment (RACDT) on clinical malaria incidence. ^1^Negative binomial analysis of monthly facility cases (random intercept for facility); adjusted for previous month’s cases, normalized difference vegetation index (NDVI), precipitation, altitude, nighttime light, number of rapid diagnostic tests done each month, and seasonality (Fourier term). Unadjusted estimate: 1.08 (95% CI: 0.78–1.49). ^2^Negative binomial difference-in-differences model (one pre- and one post-time point), adjusted for prior month’s cases, calendar month, rainfall, and EVI monthly anomalies. ^3^The 95% CI lower limit is higher here than in the published paper (rate ratio = 0.71 (95% CI: 0.22–1.20). Effect size from (nonlinear) marginal effect post-estimation from a negative binomial model with offset for cluster-level person time; variables for RDA, reactive vector control, interaction between RDA and reactive vector control, and adjusted for 2016 incidence of local cases. Unadjusted marginal effects from post-estimation (from unadjusted negative binomial model with terms for RACDT, reactive indoor residual spraying, and the interaction between the two, with offset for cluster-level person time): 0.82 (0.26–1.37). ^4^Poisson regression model adjusted for age. Unadjusted estimate from a logistic regression model (with a random effect for cluster): 1.04 (95% CI: 0.57–1.91). ^5^Negative binomial regression model of local cases with offset for person-time and adjusted for baseline (2014–2015) incidence of local cases. Unadjusted estimate: 1.06 (95% CI: 0.57–1.98).

These three trials plus three additional ones comparing RDA to no RDA were pooled to provide an estimate of the effect of RDA on clinical malaria incidence. Each trial individually showed that RDA had a null effect or nonsignificant reduction in clinical malaria incidence; a pooled analysis indicated that RDA had a nonsignificant reduction in clinical malaria incidence, adjusting for other factors (pooled RR: 0.93; 95% CI: 0.82–1.05) (Figure 6). A sensitivity analysis omitting the Eisele studies, given their different approach using four rounds of active case detection as opposed to continuous passive case detection, showed a marginally significant reduction in clinical malaria incidence (pooled OR: 0.79; 95% CI: 0.63–0.99) (Supplemental Figure 9).

One NRS (Quispe 2018^29^) used a time-series analysis of routine health facility data to report clinical malaria incidence from two districts implementing RDA and eight control districts over 2 years in Tumbes, Peru. Using a mixed-effects Poisson regression model and controlling for several environmental variables, the authors found RDA reduced the incidence of clinical malaria (RR: 0.59; 95% CI: 0.40, 0.86) (Supplemental Figure 10).

#### Parasite prevalence among the population participating in RACDT.

Two NRS (Searle 2020^27^ and Fontoura 2016^28^) assessed parasite prevalence among those receiving RACDT rather than at the community level. In the controlled before-and-after study in Brazil, households receiving RACDT, along with control households (in the same locality but >5 km from the index household), were followed up at 30, 60, and 180 days after RACDT and tested again by microscopy and PCR. In the Zambia uncontrolled before-and-after study (Searle 2020^27^), individuals in index households and their neighbors that participated in RACDT were followed up and tested again by RDT and PCR at 30 and 90 days. Results from a difference-in-differences analysis of the Brazil study indicated increases in parasite prevalence over time in RACDT households compared with control households (0.8%-points, 3.8%-points, and 2.3%-points at 30, 60, and 180 days, respectively). The Zambia study reported a decrease in parasite prevalence in RACDT households (by 0.9%-points and 2.1%-points at 30 and 90 days after RACDT, respectively) (Supplemental Table 4).

#### Elimination.

No studies reported elimination of malaria or interruption of transmission.

#### Adverse events.

The three randomized trials comparing RACDT and RDA reported on adverse events (AEs), as did the RDA trial in the Gambia (Okebe 2021^34^); however, information on AEs was typically only actively solicited from the RDA arm and not from the RACDT or comparison arm. In the Zambia trial comparing RDA using DP to RACDT using AL, 123 (6.9%) mild AEs occurred in 1,775 people treated with DP^31^; all resolved. In the Namibia trial of RDA with AL compared with RACDT with AL plus low-dose primaquine, 17 of 4,247 treated participants (0.4%) in the RDA arm experienced an AE versus 1 participant of 98 (1.0%) treated in the RACDT arm; 11 AEs were considered unrelated, six possibly related and six probably related.^6^ In Eswatini, 68 (3.8%) of 1,776 participants receiving RDA with DP experienced AEs; 54 were rated as mild and 14 as moderate; no AEs were reported from the RACDT arm.^33^ In The Gambia trial, 75 AEs (7.6%) occurred among 979 participants receiving DP in the RDA arm; 69 were considered mild and 6 moderate.^34^

### Certainty of the evidence.

**Figure 7.**
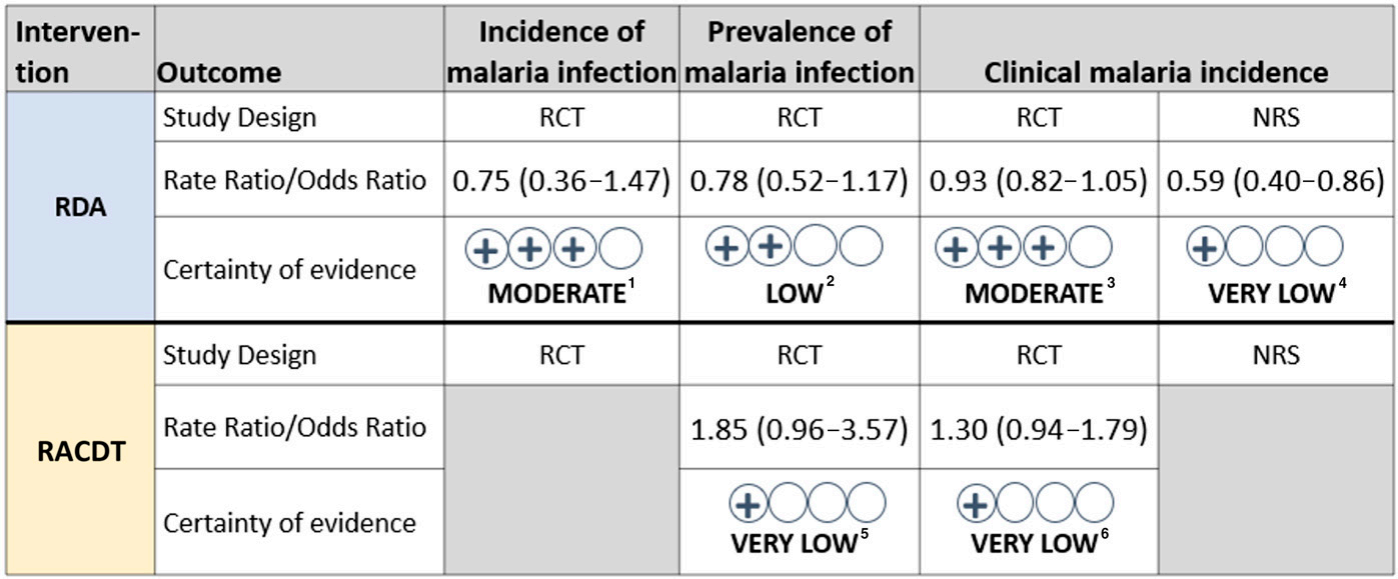
Summary of reactive case detection and treatment (RACDT) and reactive drug administration (RDA) outcomes and Grading of Recommendations, Assessment, Development, and Evaluations for each. RCT = randomized controlled trial; NRS = nonrandomized study. ^1^Rated not serious on risk of bias, inconsistency, indirectness; rated serious on imprecision. ^2^Rated not serious on risk of bias, inconsistency; rated serious on indirectness, imprecision. ^3^Rated not serious on risk of bias, inconsistency, imprecision; rated serious on indirectness. ^4^Rated serious on risk of bias; rated not serious on inconsistency, indirectness, imprecision. ^5^Rated not serious on risk of bias, inconsistency; rated very serious on indirectness; rated serious on imprecision. ^6^Rated not serious on risk of bias, inconsistency; rated very serious on indirectness; rated serious on imprecision.

After grading the certainty of evidence considering the risk of bias among included studies, their inconsistency, indirectness, imprecision, and other criteria, we found very low certainty of evidence for all RACDT outcomes given serious concerns with the indirectness of the comparison (to RDA rather than no RACDT) or serious concerns with the risk of bias and inconsistency of estimates for the NRS (Figure 7). The certainty of evidence was somewhat higher for RDA, although serious concerns with imprecision and indirectness (for some outcomes) downgraded the certainty of evidence for cRCTs to moderate or low (Figure 7); the certainty was very low from the one NRS due to high risk of bias.

### Contextual factors.

No publications were identified that reported on the values and preferences of populations with respect to the outcomes for either RACDT or RDA, nor were any studies identified that discussed the effect of RACDT or RDA on health equity. Four publications included information on acceptability, nine on costs, and 17 on various aspects of feasibility of RACDT (Supplemental Table 6). Five RDA studies in sub-Saharan Africa had associated publications reporting on contextual factors, including acceptability (*N* = 6 publications), feasibility (*N* = 6), and cost (1) (Supplemental Table 8).

#### Financial and economic considerations.

For RACDT, the cost per person screened ranged from US $5.21 in Thailand^36^ to $14.3 in Senegal^37^ to US $27.6 in Indonesia, where the general cost of screening one individual during RACDT was $11, with an additional microscopy cost of $0.62 per person and $16 per person for LAMP^38^ (Supplemental Table 7). In Indonesia, the cost per infection found by RACDT using microscopy only versus RACDT using LAMP only was US $8,930 and US $6,915, respectively. The cost per infection identified and incremental cost-effectiveness ratio for finding cases declined with increasing test positivity rate and increasing diagnostic yield.^38^ The largest cost drivers were personnel costs (41% in Indonesia^38^ and 37% in Senegal^37^), followed by training and capital costs (e.g., tablets, vehicles, and laboratory costs) (Supplemental Table 8). No detailed costing studies were conducted on RDA except for one associated with the Zambia trial (Eisele 2020—LOW^29^ and Eisele 2020—HIGH^30^); however, the costs factored into that analysis included the additional costs of the active case detection component that is not typical of RDA and, therefore, we did not include these estimates.

#### Sociocultural acceptability.

Community acceptance of RACDT was generally high,^39^^–^^42^ with refusal rates of 2% or lower. In Namibia, some “hesitation/resistance” during pretrial interviews was reported, but community engagement and sensitization improved participation (Supplemental Table 9).^42^ Similarly in Senegal, high RACDT participation was attributed to advanced cascade sensitization, making follow-up appointments for absent members, and conducting return visits to the compound the same or next day.^40^ Lack of community confidence in community health workers’ (CHWs’) ability to address diseases other than malaria and community unwillingness to visit CHWs for malaria testing were reported from Zambia.^41^

Community acceptance of RDA was similarly high (refusal rates of 2% or lower) in Namibia and Zambia.^32^^,^^43^ However, in Eswatini, the overall refusal rate was ∼4% with refusal rates of 1.4% (11/776) and 5.3% (65/1,232) in seasons 1 and 2, respectively. In Zambia, researchers attributed a large increase in self-reported acceptability (from 62% to 98%) of RDA from the baseline to follow-up survey for adults and children to intensive community-wide sensitization programs that occurred during community health worker visits.^44^ Qualitative research conducted during the RDA trials found that some participants in Namibia^42^ and The Gambia^45^ expressed concerns about taking medicine if they were not sick and skepticism about antimalarials given by CHWs in Zambia^44^; continued community sensitization has been recommended to mitigate these stigmas.

#### Feasibility and health systems considerations.

Lack of notification of malaria cases by the private sector, which limited opportunities to implement reactive strategies, was noted to be a challenge in Cambodia^46^^,^^47^ and Zanzibar (Supplemental Table 10).^48^ Within the public health sector, delayed presentation of malaria patients to health facilities, lack of malaria RDTs, complexity of case investigation procedures, and lack of standard operating procedures have been reported as barriers to effective RACDT.^49^ RDT stockouts resulted in fewer cases being investigated in Namibia^32^ and Zambia, especially during peak malaria transmission seasons in Zambia, when CHWs were overwhelmed by patient volumes.^41^

A major challenge noted in several RACDT studies was the limit of detection of RDTs and the inability of *Plasmodium falciparum–*specific RDTs to detect other species.^49^^–^^51^ To overcome these challenges, LAMP or other more sensitive diagnostics have been recommended.^49^^,^^52^

The proportion of RACDT index case households interviewed varied across geographic locations from 49% of index case households in Zanzibar to 100% in Jiangsu, China (Supplemental Table 11). Similarly, the proportion of households reached in a timely manner varied across different locations, from ∼20% in Zanzibar to 100% in China. Barriers to timely follow-up during RACDT included difficulty accessing mountainous terrains and highly mobile populations on the China–Myanmar border,^53^ flooded areas in Zambia,^41^ and the large numbers of households to screen, particularly in high-density areas of the Asia Pacific regions.^54^ In Zambia, suggestions to improve RACDT included additional CHWs or suspension of RACDT during the high-transmission season, and rain gear and access to boats for CHWs serving flood-prone areas.^41^ Lack of health workers,^49^^,^^55^ along with low motivation^41^^,^^47^ to conduct RACDT, especially during weekends or holidays, were reported in a few studies. Maintaining workforce motivation and providing consistent support, supervision and incentives have been recommended to overcome these challenges.^41^^,^^47^

Adherence to AL during RACDT was reported from one study in Namibia, which found nearly 100% adherence in 368 individuals who had their blister packs at follow-up pill counts; among individuals without their blister packs (*N* = 316), all but one reported full adherence to AL.^32^

Information on the feasibility of implementing RDA was limited to coverage and adherence data. RDA coverage (proportion of index cases followed up) varied between countries with a low of 62% in Ewsatini^33^ to ∼97% in The Gambia.^34^ RDA adherence, defined as taking all three doses of DP and verifying that no tablets were remaining in the blister pack, was above 90% in three studies in Eswatini, Gambia, and Zambia (Supplemental Table 12).^33^^,^^34^^,^^56^ However, a separate evaluation of The Gambia (Okebe 2021^34^) trial found lower adherence of 85.3% (223/273) when using examination of medicine bags and pill counts compared with self-reported adherence (91.6%; 208/227).^57^ DP was generally reported to be well tolerated with mild to moderate self-limiting side effects. However, a study from Zambia reported that 5% of patients receiving DP stopped treatment early due to unspecified side effects.^56^ In the Namibia trial, respondents anticipated reluctance about completing the full course DP, explaining that some people may save medicine to treat future illness, given distance to health facilities.^42^

## DISCUSSION

Despite the frequency with which RACDT is implemented among countries pursuing malaria elimination and the number of articles published on RACDT, no rigorous studies have assessed its impact on malaria transmission at the community level and few studies have measured any outcomes at community-level. When the impact of RACDT on malaria transmission was compared with RDA using a randomized study design, results favored RDA, although differences were not statistically significant. Given the very low certainty of the evidence available, we are unable to say whether RACDT reduces the incidence of parasitemia or clinical malaria, or the prevalence of infection.

RDA has theoretical advantages over RACDT with respect to reducing malaria transmission. Provision of a full therapeutic course of antimalarial medicine to everyone living with or near a confirmed case of malaria not only clears all existing infections but protects everyone from malaria for at least several weeks. In comparison, RACDT is likely to miss low-density infections and only those testing positive will benefit from a prophylactic period. Empirical evidence for the impact of RDA on malaria transmission is very recent and still emerging. The six randomized studies of RDA that met the eligibility criteria for this review were published between 2020 and 2022. However, only half of these trials used a true control group. Despite the theoretical advantages of RDA, the evidence available suggests that RDA probably results in little to no reduction of parasitemia incidence, may result in little to no reduction of parasite prevalence, and probably results in little to no reduction of clinical malaria incidence. By extension, despite the lack of direct evidence, RACDT is likely to have extremely limited to no impact on malaria transmission.

Evaluation of reactive malaria interventions poses several important challenges to researchers. Reactive strategies are only feasible to implement at high coverage when malaria transmission levels are low to very low, which makes it difficult to measure outcomes for statistical comparisons to be sufficiently powered to detect differences. Randomization of large units such as districts could help to overcome the challenge posed by very low levels of transmission. However, larger areas make it more difficult and expensive to conduct trials. An alternative to cRCTs could be interrupted time series designs, preferably with control areas, in locations where passive surveillance systems are sufficiently robust to measure the incidence of clinical malaria reliably. Serological markers associated with short-lived antibody responses to malaria infection may provide information on infection over a period of time, thus improving the “event” rate for outcomes in very low transmission settings.^31^ However, serological markers are not yet validated in the context of intervention trials.

Modeling studies support the findings of little to no impact of either RACDT or RDA on malaria transmission but suggest that RDA is likely to have a more favorable impact than RACDT. One modeling study using data from Zambia and simulations from an agent-based model (EMOD DTK v2.0) found that improved case management and control of imported malaria alone was sufficient to interrupt transmission in low-transmission areas, with little additional benefit from RACDT; in higher transmission settings (malaria prevalence ranging from 5% to 50%), high coverage of case management (all cases treated) and mass drug administration (MDA) had to be in place before RACDT had any appreciable effect in low population-density settings, while in higher population-density settings, RACDT did not improve chances of elimination.^58^ Another modeling study using the same Zambia data and agent-based model found that RDA (treating households within 200 m of a passively detected case) was unlikely to result in interruption of transmission (although at higher coverage levels, MDA or RDA based on exposed households identified through serological markers were both likely to contribute to elimination); in settings of higher malaria prevalence and intervention coverage, RDA strategies were just as effective as MDA at reducing onward transmission.^59^

Despite the lack of evidence for their effectiveness, community acceptance of both RACDT and RDA appears to be high, with refusal rates typically less than 2% and reports of high levels of adherence to antimalarial medicines among those treated or provided with medicines. Strong sensitization efforts were found to encourage participation for both strategies and were critical for high acceptance rates. Reactive strategies can be time-consuming, however, and several programs faced challenges in following up each index case when caseloads were higher. They also must be sustained, otherwise any gains in transmission reduction are lost; however, at very low transmission levels, this activity should be sustainable (and decrease over time). An additional challenge with RACDT in particular is the relatively low sensitivity of RDTs for detecting low-density, afebrile malaria infections. The average cost per-person screened using RDTs ranged from about $5^37^ to $12.^60^ Not surprisingly, the cost per secondary case identified tended to decline with increasing test positivity rates and diagnostic yield.^37^ Cost data for RDA were extremely limited, and the only costing study was from two studies in Zambia and included the cost of active case detection, which is typically not part of RDA.

The few studies on RACDT and RDA found through the literature search precluded examination of potential effect modifiers identified a priori. However, several of these factors could improve the effectiveness both strategies. For example, reactive strategies that occur around an imported case are likely to have less of an impact than those around indigenous cases. The type of antimalarial medicine used in RDA and the length of its prophylactic period will determine how protective the intervention is likely to be. Coverage has been shown to be an important factor in most interventions but is rarely measured in reactive strategies, and little is known about its effect. The radius of the intervention response around a confirmed case is highly variable between malaria programs, and there is no evidence on what the optimal distance would be. Finally, although some malaria programs may attempt to implement reactive strategies in nonelimination settings, the higher rate of transmission and increased levels of immunity in the population are likely to decrease the effectiveness of the strategy.

Countries pursuing elimination or aiming to reduce already-low transmission are increasingly implementing reactive strategies such as RACDT and RDA despite the extremely limited evidence base. From the three trials comparing RACDT with RDA, it appears that RDA might be more effective than RACDT for reducing malaria transmission, although evidence generally is of low certainty and differences are not statistically significant. This is likely due to several factors, including the treatment of low-density infections not detected by RDTs, the prophylactic effect of treating noninfected individuals, and the use of longer acting antimalarials (DP versus AL). Given limited financial and human resources, countries with low malaria transmission face difficult choices about whether to implement these reactive drug-based strategies.

## Supplemental Materials

10.4269/ajtmh.22-0720 Supplemental Materials
