# Supplementary material for: Reactive Case Detection and Treatment and Reactive Drug Administration for Reducing Malaria Transmission: A Systematic Review and Meta-Analysis
Source: Am J Trop Med Hyg. 2023 Dec 20;110(4 Suppl):82–93. doi: 10.4269/ajtmh.22-0720 (PMC10993791; doi:10.4269/ajtmh.22-0720)
Supplement: Supplemental Materials [file tpmd220720.SD1.pdf]

Supplemental materials

Table S1: Search terms and results

Search Query: Reactive strategies include either: reactive case detection (testing and treating those positive) around an index case (presenting to health facilities or community health workers), drug administration (without testing) for households around an index case

Search Strategy:

Database	Strategy	Run Date	Records
**Medline (OVID) 1946-**	Malaria*<br>AND<br>(((screen* ADJ5 treat*) OR (test* ADJ5 treat*)) AND (focal OR foci OR index OR contact*)) OR (reactive* ADJ5 detect*) OR (passive* ADJ5 detect*) OR (reactive* ADJ5 administration) OR (reactive* ADJ5 screen*) OR (reactive* ADJ5 test*) OR (reactive* ADJ5 treat*) OR (focal ADJ5 administration) OR (foci ADJ5 administration) OR (focal ADJ5 MDA) OR (foci ADJ5 MDA)	11/16/2020	416
**Embase (OVID) 1988-**	Malaria*<br>AND<br>(((screen* ADJ5 treat*) OR (test* ADJ5 treat*)) AND (focal OR foci OR index OR contact*)) OR (reactive* ADJ5 detect*) OR (passive* ADJ5 detect*) OR (reactive* ADJ5 administration) OR (reactive* ADJ5 screen*) OR (reactive* ADJ5 test*) OR (reactive* ADJ5 treat*) OR (focal ADJ5 administration) OR (foci ADJ5 administration) OR (focal ADJ5 MDA) OR (foci ADJ5 MDA)<br>NOT pubmed/medline	11/16/2020	715<br>-387 duplicates<br>=328 unique items
**Global Health (OVID) 1910_**	Malaria*<br>AND<br>(((screen* ADJ5 treat*) OR (test* ADJ5 treat*)) AND (focal OR foci OR index OR contact*)) OR (reactive* ADJ5 detect*) OR (passive* ADJ5 detect*) OR (reactive* ADJ5 administration) OR (reactive* ADJ5 screen*) OR (reactive* ADJ5 test*) OR (reactive* ADJ5 treat*) OR (focal ADJ5 administration) OR (foci ADJ5 administration) OR (focal ADJ5 MDA) OR (foci ADJ5 MDA)	11/16/2020	451<br>-334 duplicates<br>=117 unique items
**Cochrane Library**	Malaria*:ti,ab<br>AND<br>((((screen* NEAR/5 treat*) OR (test* NEAR/5 treat*)) AND (focal OR foci OR index OR contact*)) OR (reactive* NEAR/5 detect*) OR (passive* NEAR/5 detect*) OR (reactive* NEAR/5 administration) OR (reactive* NEAR/5 screen*) OR (reactive* NEAR/5 test*) OR (reactive* NEAR/5 treat*) OR (focal NEAR/5 administration) OR (foci NEAR/5 administration) OR (focal	11/16/2020	161<br>-105 duplicates<br>=56 unique items

	NEAR/5 MDA) OR (foci NEAR/5 MDA)):ti,ab		
**CINAHL (EbscoHost)**	Malaria*<br>AND<br>((((screen* N5 treat*) OR (test* N5 treat*)) AND (focal OR foci OR index OR contact*)) OR (reactive* N5 detect*) OR (passive* N5 detect*) OR (reactive* N5 administration) OR (reactive* N5 screen*) OR (reactive* N5 test*) OR (reactive* N5 treat*) OR (focal N5 administration) OR (foci N5 administration) OR (focal N5 MDA) OR (foci N5 MDA))	11/16/2020	75<br>-57 duplicates<br>=18 unique items
**Scopus**	TITLE-ABS-KEY(Malaria*) AND TITLE-ABS-KEY((((screen* W/5 treat*) OR (test* W/5 treat*)) AND (focal OR foci OR index OR contact*)) OR (reactive* W/5 detect*) OR (passive* W/5 detect*) OR (reactive* W/5 administration) OR (reactive* W/5 screen*) OR (reactive* W/5 test*) OR (reactive* W/5 treat*) OR (focal W/5 administration) OR (foci W/5 administration) OR (focal W/5 MDA) OR (foci W/5 MDA))	11/16/2020	552<br>-445 duplicates<br>=107 unique items
**Clinicaltrials.gov**	Malaria | reactive case detection | completed<br>OR<br>focal mass drug administration OR foci mass drug administration OR focal MDA OR foci MDA | Completed Studies | malaria	11/16/2020	6<br>-1 duplicates<br>=5 unique items
**Global Index Medicus**	Malaria*<br>AND<br>((screen* OR test*) AND (focal OR foci OR index OR contact*)) OR (reactive* AND detect*) OR (passive* AND detect*) OR (reactive* AND administration) OR (reactive* AND screen*) OR (reactive* AND test*) OR (reactive* AND treat*) OR (focal AND administration) OR (foci AND administration) OR (focal AND MDA) OR (foci AND MDA)	11/16/2020	313<br>-36 duplicates<br>=277 unique items

Notes: Duplicates were identified using the Endnote automated "find duplicates" function with preference set to match on title, author and year, and removed from your Endnote library. There will likely be additional duplicates found that Endnote was unable to detect.

Table S2: Potential effect modifiers considered for sub-group analyses

Primary effect modifiers (pre-specified sub-group analysis)	Additional effect modifiers to be collected
• Level of transmission[1]<br>• Vector control coverage<br>• Malaria parasite species (Pf, Pv, Po or Pm) of index cases<br>• Antimalarial medication used, including gametocytocide or hypnozoiticide<br>• Size or population of intervention area (e.g., radius) around the confirmed case<br>• Use of symptom screening before testing (RACDT only)<br>• Limits of detection and sensitivity of the test (RACDT only)	• Whether all cases were included or only cases classified as local<br>• Coverage of the intervention<br>• Availability of G6PD screening (for Pv areas)<br>• Rural vs. urban area

[1] The level of transmission was categorized according to the following schema found in the *Framework for malaria elimination:* High: incidence of about 450/1000 or Pf prevalence of >=35%; Moderate: incidence of 250-450 per 1000 and Pf/Pv prevalence of 10-35%; Low: incidence of 100-250 per 1000 and Pf/Pv prevalence of 1-10%; Very low: incidence of <100 per 1000 and Pf/Pv prevalence <1%.

Table S3: Descriptions of included studies

Both RACDT and RDA

Randomized studies

Bridges 2021

Methods

Location: Southern Province, Zambia

Study dates: May 2016 – May 2018

Baseline annual parasite incidence: ~3 per 1,000

Study design: Cluster-randomized trial

Unit of randomization: Health facility catchment area

Total number of clusters (total): 16

Clusters in RACDT and RDA arms: 8 RACDT and 8 in RDA arm

Total population in RACDT and RDA arms: ~56,000 in RACDT arm; ~63,000 in RDA arm

Total population in control/comparison arm: ~56,000

Participants

	RACDT	RDA
Number of 'events'	392	302
Number of household members/neighbors tested:	3,953	N/A
Number of household members/neighbors treated:	118	1,775
Percent treated of total targeted: N/A	N/A	95.2% (1,775/1,865)

Interventions

	RACDT	RDA
Index case detection	Passive surveillance at health facilities and CHWs	
Drug used for household members/neighbors	Artemether-lumefantrine	Dihyroartemisinin-piperaquine
Area targeted around index case	140 meters	140 meters
Co-interventions	ITNs, IRS, good malaria case management	

Outcomes

<u>Prevalence of parasitemia</u>:

	Measurement: Cross-sectional household survey<br>Persons sampled: Children aged ≥1 month to <15 years<br>Diagnostic test used: PCR<br>Time point(s): One time post-intervention (April–May 2018)<br>Sample size: 3,151 (RDA), 3,125 (RACDT)<br><u>Incidence of clinical cases:</u><br>Measurement: Weekly and monthly routine data on malaria cases (clinical and laboratory-confirmed) from community health workers and facilities accessed through DHIS2<br>Time points: May 2016 – May 2018<br><u>Adverse events:</u><br>No adverse events reported from RACDT arm (using AL). Dihyroartemisinin-piperaquine used in RDA arm; artemether-lumefantrine used in RACDT arm. 123 reported only in RDA arm: headache (20%), abdominal pain (17%), dizziness (17%), or nausea (16%). All were mild and self-resolved.

Risk of bias

**Bias**	**Authors' judgement**	**Support for judgement**
Randomization process	Low risk	Clusters were picked from a hat.
Recruitment of participants into clusters	Some concerns	Authors speculated there was a higher refusal rate in RDA arm.
Deviations from intended interventions	Low risk	No evidence that incorrect interventions delivered.
Missing outcome data (clinical malaria incidence)	Low risk	Authors reported no missing routine data (per correspondence)
Missing outcome data (adverse events)	High risk	Adverse events reported only in RDA arm
Measurement of outcome (clinical malaria incidence)	Low risk	Data collected at health facilities and by CHWs for clinical management.
Measurement of outcome (adverse events)	High risk	Unclear whether adverse events reported from non-RDA arm
Selection of reported result (clinical malaria incidence)	Some concerns	Although outcome was pre-specified, the pre-specified model in the published protocol only mentioned adjustment for environmental variables, but not for previous month's cases and RDTs done, which were both included.
Selection of reported result (adverse events)	Low risk	Standard adverse events reported.

Hsiang 2020

Methods	Location: Zambezi region, northern Namibia<br><br>Study dates: January–December 2017<br><br>Baseline annual parasite incidence: 32.5 per 1,000 in 2016 (previously <15 per 1,000 since 2010)<br><br>Study design: Cluster-randomized trial with factorial design: RDA, reactive vector control, both, and RACDT (treated as the control)<br><br>Unit of randomization: Census enumeration area<br><br>Total number of clusters (total): 56<br><br>Clusters in RACDT and RDA arms: 28 in RACDT (14 also with reactive IRS)) and 28 in RDA (14 also with reactive IRS)<br><br>Total population in RACDT and RDA arms: ~16,500 in each arm (total population in catchment areas = 33,418)
Participants	

	RACDT	RDA
Number of 'events'	178	164 (from 492 eligible cases)
Number of household members/neighbors tested:	4,701 (88.7% of total targeted)	N/A
Number of household members/neighbors treated:	98 (of 114 testing positive)	4,247
Percent treated of total targeted: N/A	86.0%	86.7%

Interventions

	RACDT	RDA
Index case detection	Passive surveillance at health facilities	
Drug used for household members/neighbors	Artemether-lumefantrine plus low-dose (0.25 mg/kg) primaquine	Artemether-lumefantrine
Area targeted around	500 meters	500 meters

<table>
<tr><td></td><td><table>
<tr><td>index case</td><td></td><td></td></tr>
<tr><td>Co-interventions</td><td colspan="2">Case management, annual IRS with DDT; reactive-IRS in half the RACDT and half the RDA clusters</td></tr>
</table></td></tr>
<tr><td>Outcomes</td><td><u>Prevalence of parasitemia</u>:

Measurement: Cross-sectional household survey

Persons sampled: All household members

Diagnostic test used: PCR

Time point(s): May – August 2017

Sample size: 2,150 (RACDT), 1,932 (RDA)

Analysis: Authors used a log binomial regression with a log link to estimate prevalence ratios using generalized estimating equations to adjust for enumeration area-level clustering. Models included terms for RDA, for reactive IRS, and the interaction between the two. Adjusted model also included 2016 incidence of local cases, index case level and target population coverage for RAD or RDA, response times, and co-interventions by the Ministry of Health.

<u>Incidence of clinical cases:</u>

Measurement: Routine data on malaria cases diagnosed by microscopy and RDT at health facilities

Time points: Jan 2017 – December 2017

Analysis: Negative binomial regression using a generalized linear model to estimate incidence rate ratios using cluster-level case data and cluster person-time as an offset. Models included terms for RDA, for reactive IRS, and the interaction between the two. Adjusted model also included 2016 incidence of local cases, index case level and target population coverage for RAD or RDA, response times, and co-interventions by the Ministry of Health. Note that cases and person-time counted starting only 8 weeks after the first intervention administered in each cluster.

<u>Adverse events:</u>

It is unclear how AEs were detected in the RACDT arm. AEs in RDA arm were detected by having participants call an on-call study nurse and by follow-up visits by a study nurse among a portion of those receiving artermether-lumefantrine. In total 23 AEs in 18 individuals were reported, including headache (n=5), dizziness (n=5), diarrhea (n=3), vomiting (n=2), abdominal pain (n=2), fever (n=2), and weakness, cough, decreased appetite and muscle pain (1 each); 19 (83%) AEs were mild (grade 1) and four (17%) were moderate (grade 2). 17 (74%) of 23 adverse events were actively detected at follow-up visits. Six AEs were classified as probably related to treatment, 6 as possibly related, and 11 as unrelated. Of the 18 participants reporting at least one AE, 17 were in the RDA group and 1 in</td></tr>
</table>

	the RACDT group.

Risk of bias

**Bias**	**Authors' judgement**	**Support for judgement**
Randomization process	Low risk	Computer-generated restricted randomization used
Recruitment of participants into clusters	Low risk	Unlikely to have been differential recruitment by arm; participation rates similar across arms
Deviations from intended interventions	Low risk	No evidence of wrong intervention or deviations due to trial context
Missing outcome data (parasitemia prevalence)	Low risk	Equal numbers of children per arm sampled; no evidence of differential missingness
Missing outcome data (clinical malaria incidence)	Low risk	Based on routine data reported; no evidence of differential missingness
Missing outcome data (adverse events)	Some concerns	Unclear to what extent adverse events were captured in the RACDT arm (although one adverse event reported from RACDT arm).
Measurement of outcome (parasitemia prevalence)	Low risk	PCR done in the laboratory; unlikely that analysis affected by knowledge of study arm
Measurement of outcome (clinical malaria incidence)	Low risk	Routine diagnosis at health facilities; unlikely that diagnosis affected by knowledge of study arm
Measurement of outcome (adverse events)	High risk	Likely that adverse event reporting was much stronger in RDA arm compared to reactive case detection arm.
Selection of reported result (parasitemia prevalence)	Low risk	Pre-stated outcome
Selection of reported result (clinical malaria incidence)	Low risk	Pre-stated outcome
Selection of reported result (adverse events)	Low risk	Pre-stated outcome

Vilakati 2021

Methods	Location: eastern malaria-endemic areas of Eswatini<br>Study dates: September 2015 – August 2017<br>Baseline annual parasite incidence: 5.2 per 1,000 from 2012–2015<br>Study design: Cluster-randomized trial comparing RDA to RACDT

<table>
<tr><td></td><td>Unit of randomization: Locality

Total number of clusters (total): 77

Clusters in RACDT and RDA arm: 39

Total population in RACDT and RDA arms: 2,752 in RACDT; 2,680 in RDA (note that the populations for both RACDT and RDA only included the ‘at-risk’ population (part of the enumeration area within the locality that had incident cases during the trial))</td></tr>
<tr><td>Participants</td><td>
<table>
<tr><th></th><th>RACDT</th><th>RDA</th></tr>
<tr><td>Number of ‘events’</td><td>46 (covering 53 cases (of 99 reported) in 22 localities)</td><td>64 (covering 76 cases in 25 localities)</td></tr>
<tr><td>Number of household members/neighbors tested:</td><td>1,455 (78.4% of total targeted)</td><td>N/A</td></tr>
<tr><td>Number of household members/neighbors treated:</td><td>5 (of 5 testing positive)*</td><td>1,776</td></tr>
<tr><td>Percent treated of total targeted: N/A</td><td>100% (5/5)</td><td>72.3%</td></tr>
</table>
* Those testing positive were referred to nearest health facility for treatment.</td></tr>
<tr><td>Interventions</td><td>
<table>
<tr><th></th><th>RACDT</th><th>RDA</th></tr>
<tr><td>Index case detection</td><td colspan="2">Passive surveillance at health facilities</td></tr>
<tr><td>Drug used for household members/neighbors</td><td>Artemether-lumefantrine</td><td>Dihyroartemisinin-piperaquine</td></tr>
<tr><td>Area targeted around index case</td><td>500 meters</td><td>200 meters (minimum 30 individuals)</td></tr>
<tr><td>Co-interventions</td><td colspan="2">Case management, pre-season IRS</td></tr>
</table></td></tr>
<tr><td>Outcomes</td><td><u>Incidence of clinical cases:</u>

Measurement: Routine data on malaria cases diagnosed by microscopy and RDT at health facilities. Only local cases included in outcome (although the authors did an analysis with all cases as well).

Time points: Jan 2017 – December 2017</td></tr>
</table>

	Analysis: Negative binomial regression with an offset for cluster population size. The first index case in each cluster was not included to allow time for the intervention to have an effect. Adjusted model included covariates associated with an outcome (not pre-specified), which was incidence of local cases in 2014 – 2015.<br><br><u>Adverse events</u>:<br><br>For those in the RACDT arm referred to facilities to take AL, there was not systematic counseling on AE reporting. In the RDA arm, AEs occurred in 68 individuals and were mostly headache, nausea/vomiting, and abdominal pain; 54 (80%) were mild and 14 (21%) were moderate. Counseling on AE reporting occurred during administration of dihyroartemisinin-piperaquine to those receiving RDA; there was a study nurse on call 24 hours per day, 7 days per week and also active pharmacovigilance in the community.
Notes	*RDA was largely conducted by the study staff whereas RACDT relied more on the Ministry of Health routine response.*

Risk of bias

**Bias**	**Authors' judgement**	**Support for judgement**
Randomization process	Some concerns	Baseline imbalances due to inclusion of only clusters with an index case
Recruitment of participants into clusters	Low risk	Similar participation rates in both arms of the trial
Deviations from intended interventions	Some concerns	20 RACDT interventions delivered in RDA arm (14 clusters); 5 RDA interventions delivered in RACDT arm
Missing outcome data (clinical malaria incidence)	Low risk	No reason to believe there was differential missingness by study arm
Missing outcome data (adverse events)	High risk	Adverse events reported only from RDA arm
Measurement of outcome (clinical malaria incidence)	Low risk	Routine diagnosis at health facilities; unlikely that diagnosis affected by knowledge of study arm
Measurement of outcome (adverse events)	High risk	Adverse events reported only from RDA arm
Selection of reported result (clinical malaria incidence)	Low risk	Pre-specified outcome
Selection of reported result (adverse events)	Low risk	Pre-specified outcome

RACDT Only

Non-randomized studies

Fortouna 2016

Methods	Location: Acrelandia, Acre State, Amazonia, Brazil<br><br>Study dates: January – July 2013<br><br>Baseline annual parasite incidence: 5 per 1,000 in 2012; *P. vivax* accounts for 84% cases in the area<br><br>Study design: Non-randomized before-and-after study<br><br>Population in RACDT area: 14,120<br><br>*Note: Passively-detected index cases triggered an RACDT response with household members and neighbors within a defined radius <u>plus</u> 5 randomly selected control households residing in same locality but at least 5 km from the index case. All index case, neighboring and control households were followed up at 30, 60, 90, and 180 days and all household members tested for parasitemia.*
Participants	Number of RACDT 'events': 41 (all *P. vivax*)<br><br>Number of household members/neighbors tested: 878 from index/neighboring households; 841 from control households<br><br>Percent tested of total targeted: N/A<br><br>Number of household members/neighbors positive (on Day 0): 17 of 835 (2.0%) by microscopy; 59 of 812 (7.2%) by PCR<br><br>Number of household members/neighbors treated: 5 (referred for treatment): 17 on Day 0<br><br>*Note that there was only 1 positive microscopy test among 634 (0.2%) control households tested on Day 0 by microscopy and 35 of 631 (5.5%) positive by PCR on Day 0.*
Interventions	<u>Intervention:</u><br><br>Drug(s) used for RACDT: Chloroquine (total dose: 25 mg/kg over 3 days) and primaquine (0.5 mg/kg/day for 7 days).<br><br>Index case detection: Through passive surveillance at health facilities and confirmed microscopy<br><br>Area targeted around index case: 5 nearest houses within a radius of up to 3 km<br><br>Detection of positives around index case: Thick smear microscopy (PCR also done but PCR positive/microscopy negative were not treated)<br><br><u>Co-interventions:</u> Selective IRS implemented in early 2008, widespread distribution of LLINs since 2010
Outcomes	<u>Prevalence of parasitemia among those receiving the intervention:</u><br><br>Measurement: Thick smear microscopy and PCR<br><br>Time points: On Day 0 (initial RACDT), Day 30, Day 60, and Day 180

	Details: Diagnostics were done at intervention (index case and neighbor) households as well as control households during the four time points of the study. Participants were treated if they were microscopy positive.

**Bias**	**Authors' judgement**	**Support for judgement**
Application of appropriate eligibility criteria	Low risk	Clear criteria were specified for control households (same locality, >5 kilometers from index case)
Flawed measurement in the exposure (i.e. intervention)	Low risk	Since the outcome is only among receiving the intervention, this domain is less relevant
Flawed measurement in the outcome	Low risk	Unlikely that those doing microscopy or PCR in the lab were aware of participant's status
Failure to adequately control for confounding	Low risk	Control households were included in measurement at baseline and all follow-up time points
Incomplete follow-up (loss that could introduce bias)	Low risk	There was equally good follow-up over time in the RACDT and the control households
Downgrade from low to very low?	No	

Searle 2020

Methods	Location: Macha Hospital, Choma district, Southern Provice, Zambia<br><br>Study dates: March 2016 – March 2018<br><br>Baseline annual parasite incidence: 1% PfPR in 2013<br><br>Study design: Uncontrolled before-and-after study<br><br>Population in RACDT area: 14,120<br><br>*Note: Passively-detected index cases triggered an RACDT response with household members and neighbors within 250 meters; these households with an RACDT response on Day 0 were followed up at Day 30 and Day 90 and residents tested at each time point.*
Participants	Number of RACDT 'events': 84 index cases with Day 0 visit<br><br>Number of household members/neighbors tested: 2,215 on Day 0 (676 from index households, 675 from neighbor households within 140 meters, and 864 within neighbor households within 141–250 meters<br><br>Percent tested of total targeted: N/A<br><br>Number of household members/neighbors positive (on Day 0): 26 (1.2%)

	of 225 by RDT on Day 0; 83 (3.7%) of by PCR (Pf only) on Day 0<br><br>Number of household members/neighbors treated: N/A
Interventions	Intervention:<br><br>Drug(s) used for RACDT: Artemether-lumefantrine<br><br>Index case detection: Through passive surveillance at health facilities<br><br>Area targeted around index case: 250 meters (increased from 140 in routine RACDT response)<br><br>Detection of positives around index case: RDT (HRP2)<br><br>Co-interventions: ITNs
Outcomes	Prevalence of parasitemia among those receiving the intervention:<br><br>Measurement: Thick smear microscopy and PCR<br><br>Time points: On Day 0 (initial RACDT), Day 30, and Day 90<br><br>Details: Diagnostics were done at intervention (index case and neighbor) households during the three time points of the study. Participants were treated if they were RDT positive.

Bias	Authors' judgement	Support for judgement
Application of appropriate eligibility criteria	Low risk	The RACDT strategy had been carried out previously by community health workers who had experience in identifying cases for RACDT
Flawed measurement in the exposure (i.e. intervention)	Low risk	Since the outcome is only among receiving the intervention, this domain is less relevant
Flawed measurement in the outcome	Some concerns	It is possible (but unlikely) that those doing RDTs or PCR in the lab were aware that all these participants received RACDT
Failure to adequately control for confounding	High risk	There was no comparison group leaving this study very vulnerable to secular trends
Incomplete follow-up (loss that could introduce bias)	Low risk	High rates of follow-up at two later time points
Downgrade from low to very low?	Yes	Lack of comparison group

RDA Only

Randomized

Eisele 2020-LOW and Eisele 2020-HIGH

Methods	Location: Southern Province, Zambia<br>Study dates: May 2014 – May 2016<br>Baseline annual parasite incidence: N/A; half clusters in higher prevalence (≥10% *Pf*Pr) areas; half clusters in lower prevalence (<10% *Pf*Pr) areas<br>Study design: Cluster-randomized trial<br>Unit of randomization: Health facility catchment area<br>Total number of clusters (total): 20*<br>Clusters in RDA arm: 10<br>Total population in RDA arm: ~110,000<br>Total population in control/comparison arm: ~110,000
Participants	Number of RDA 'events': Not available<br>Number of household members/neighbors treated: 65,319 over four rounds<br>Percent of total targeted: N/A but estimated household coverage = 71.4% (95% CI: 66.7, 76.) across all four rounds
Interventions	<u>Intervention:</u><br>Drug(s) used for RDA: Dihyroartemisinin-piperaquine<br>Index case detection: Through active surveillance that took place during four "rounds": 1) December 2014, 2) Feb–March 2015, 3) October 2015, and 4) February 2016; all household members were tested with a *Pf* RDT and if anyone tested positive, the entire household was treated<br>Area targeted around index case: Index case household only<br><u>Comparison:</u><br>Type: No reactive treatment (enhanced intervention package only)<br><u>Co-interventions:</u> Scaled intervention package throughout trial areas included ITNs, IRS (2 rounds with Actellic in 2014 and 2015), enhanced malaria case management through expansion of CHW-based community case management, high-quality surveillance and reporting<br><br>*Note: 20 additional health facility clusters randomized to mass drug administration (MDA) but not included in this review*.
Outcomes	<u>Incidence of parasitemia:</u><br>Cohort of targeted 2,250 individuals ≥3 months followed monthly (RDT and PCR)

	Analysis: Random-effects negative binomial regression model (with a random effect at individual and cluster level); adjusted model included PCR infection at baseline, age, gender, wealth quintile, household IRS at baseline, elevation, mean rainfall over study period, and mean environmental vegetation index over study period.<br><br>Prevalence of parasitemia:<br><br>Measurement: Cross-sectional household surveys<br><br>Persons sampled: Children aged 3 to 70 months<br><br>Diagnostic test used: RDT<br><br>Time point(s): 3 surveys conducted: 1) Pre-intervention (April–May 2014), 2) Post-intervention 1 (April–May 2015, after treatment rounds 1 and 2), and 3) Post-intervention 2 (April–May 2016, after treatment rounds 3 and 4)<br><br>Sample size (range): 304–521 (RDA), 332–505 (control)<br><br>Analysis: Logistic regression with random effect for cluster; adjusted for child age, gender, household wealth, rainfall, enhanced vegetation index (EVI), household elevation, and household protection by LLINs and IRS.<br><br>Incidence of clinical cases:<br><br>Measurement: Routine data on malaria cases from community health workers and facilities accessed through DHIS2<br><br>Time points: Jan 2012 – May 2016<br><br>Analysis: Negative binomial difference-in-differences model with random effect for cluster; adjusted for monthly total rainfall, EVI, and previous month's case counts.

Risk of bias

Bias	Authors' judgement	Support for judgement
Randomization process	Low risk	Random allocation via computer algorithm
Recruitment of participants into clusters	Low risk	No serious baseline imbalances, coverage of RDA >70%
Deviations from intended interventions	Low risk	No evidence of deviations from intended intervention.
Missing outcome data (parasitemia incidence)	Low risk	Mean monthly follow-up similar across study arms, nearly 90% completed at least 12 months of follow-up.
Missing outcome data (parasitemia prevalence)	Low risk	Separate sampling done at each survey round; no evidence of high missingness.
Missing outcome data	Low risk	Based on routine reporting of data

(clinical malaria incidence)		from facilities and community health workers in a well-established system with low missing data
Measurement of outcome (parasitemia incidence)	Low risk	PCR done in the lab and unlikely that laboratory scientists knew which cluster patients were from.
Measurement of outcome (parasitemia prevalence)	Low risk	Unlikely that study team differentially interpreted RDT results based on study cluster
Measurement of outcome (clinical malaria incidence)	Low risk	Based on routine reporting of data from facilities and community health workers.
Selection of reported result (parasitemia incidence)	Low risk	Based on previously specified analysis plan.
Selection of reported result (parasitemia prevalence)	Low risk	Based on previously specified analysis plan.
Selection of reported result (clinical malaria incidence)	Low risk	Based on previously specified analysis plan.

Okebe 2021

Methods	Location: The Gambia (North Bank East and Lower River health regions)<br>Study dates: August 2017 – December 2018<br>Baseline annual parasite incidence: Not available but malaria prevalence by molecular methods in 2012 was 4.6% and 9.4% in North Bank and Lower River regions, respectively.<br>Study design: Cluster-randomized trial of RDA compared to RACDT only for symptomatic household members of index case.<br>Unit of randomization: Village<br>Total number of clusters (total): 50 (16 villages added in November 2017 due to lower-than-anticipated malaria prevalence in the control arm)<br>Clusters in RDA arm: 25 (7 added in year 2 of trial)<br>Total population in RDA arm: 8,645<br>Total population in control/comparison arm: 10,300
Participants	Number of RDA ‘events’: 71<br>Number of household members/neighbors treated: 979<br>Percent of total targeted: 96.6%

Interventions	<u>Intervention:</u><br><br>Drug(s) used for RDA: Dihyroartemisinin-piperaquine<br><br>Index case detection: Passive detection by village health workers by RDT<br><br>Area targeted around index case: Compound of index case (all residents)<br><br><br><u>Comparison:</u><br><br>Type: Reactive case detection with artemether-lumefantrine for symptomatic members of the index case compound who tested positive by RDT.<br><br><u>Co-interventions:</u> Not specified
Outcomes	<u>Prevalence of parasitemia</u>:<br><br>Measurement: Cross-sectional survey (done twice, in 2017 and 2018) with finger-prick blood collection for PCR. The 2018 survey results were used for this review.<br><br>Persons sampled: Random sample (proportional to village size) of residents of all ages<br><br>Diagnostic test used: PCR<br><br>Time point(s): 2017 and 2018<br><br>Sample size: In 2018: 1,924 (RDA) and 1,824 (control)<br><br>Analysis: Random effects logistic regression model; adjusted model included age.<br><br><u>Incidence of clinical cases:</u><br><br>Measurement: Routine data on malaria cases diagnosed by microscopy and RDT at health facilities<br><br>Time points: Jan 2017 – December 2017<br><br>Analysis: Random effects logistic regression model; adjusted model included age.<br><br><u>Adverse events:</u><br><br>Village health workers (who delivered the RDA) returned on day four to ask about adverse events; it is unclear if/how AEs were solicitated in RACDT arm. Total of 75 AEs among the 979 participants receiving dihyroartemisinin-piperaquine: 11 (14.7%) vomiting, 10 (13.3%) loose stools, 7 (9.3%) diarrhea, 7 (9.3%) dizziness (7 (9.3%) nausea and the rest included body aches, abdominal pain headache, tiredness, weakness, and other. 69 AEs considered mild and 6 moderate.

Risk of bias

Bias	Authors' judgement	Support for judgement

Randomization process	Low risk	Computer-generated algorithm by trial statistician
Recruitment of participants into clusters	Low risk	Confirmed cases fully investigated in both RDA and RACDT arms
Deviations from intended interventions	Low risk	High participation and adherence to treatment in RDA arm
Missing outcome data (parasitemia prevalence)	Low risk	No evidence of differential missingness by study arm
Missing outcome data (clinical malaria incidence)	Low risk	Based on routine data reported; no evidence of differential missingness
Missing outcome data (adverse events)	High risk	Adverse events reported only from RDA arm
Measurement of outcome (parasitemia prevalence)	Low risk	Random sample of participants drawn from each village in each study arm
Measurement of outcome (clinical malaria incidence)	Some concerns	Routine diagnosis by village health workers who also delivered intervention; unlikely but possible that diagnosis affected by knowledge of study arm
Measurement of outcome (adverse events)	High risk	Active follow-up for adverse events only in RDA arm
Selection of reported result (parasitemia prevalence)	Low risk	Pre-specified outcome
Selection of reported result (clinical malaria incidence)	Low risk	Pre-specified outcome
Selection of reported result (adverse events)	Low risk	Pre-specified outcome

Non-randomized studies

Quispe 2018

Methods	Location: Tumbes region of Peru<br>Study dates: 2009 (month unspecified) to 2010 (month unspecified)<br>Baseline annual parasite incidence: 8.2 per 1,000 in 2010 (almost all *P. vivax*)<br>Study design: Non-randomized quasi-experimental study using weekly malaria incidence data<br>Total population in RDA arm: 36,231 (2 districts)<br>Total population in comparison area: 163,984 (8 districts)
Participants	Number of RDA 'events': 867

	Number of household members/neighbors treated: 7,376<br>Percent of total targeted: Not available
Interventions	Intervention:<br>Drug(s) used for RDA: CQ (25 mg/kg) for 72 hours plus PQ (0.5 mg/kg) for 7 days<br>Index case detection: Through passive surveillance at health facilities and confirmed by microscopy<br>Area targeted around index case: Only household members plus social contacts and excluding children <5, adults >65, pregnant women, chronically ill<br>Comparison:<br>Type: Routine passive case detection at health facilities; diagnosis by microscopy, and treatment with CQ (25 mg/kg) for 72 hours plus PQ (0.5 mg/kg) for 7 days for positive cases.<br>Co-interventions: Not specified
Outcomes	Incidence of clinical cases:<br>Measurement: Routine weekly data on malaria cases diagnosed by microscopy at health facilities.<br>Time points: 2009 –2010<br>Analysis: Mixed effects Poisson regression of weekly cases with variable for intervention district. Adjusted model included climate covariates associated with outcome, including pressure, humidity, temperature, moisture, precipitation , and vegetation.<br>Adverse events:<br>None reported from any of the 13 districts (2 study plus 11 comparison) in the study.

Risk of bias

Bias	Authors' judgement	Support for judgement
Application of appropriate eligibility criteria	Some concerns	The two intervention districts had substantially higher malaria transmission at baseline than those included in the control
Flawed measurement in the exposure (i.e. intervention)	Some concerns	No information on the proportion of cases that were followed up with RDA
Flawed measurement in the outcome	Some concerns	No information was provided on the coverage of health systems in the intervention and control communities
Failure to adequately	Sine concerns	Differences in baseline risk between

control for confounding		intervention and comparison districts do not seem to be accounted for in the analysis model.
Incomplete follow-up (loss that could introduce bias)	Low risk	No reason to expect that that there was loss to follow up as the indicator for malaria incidence is at the cluster level.
Downgrade from low to very low?	Yes	

Table S4: Parasite prevalence by PCR among those receiving RACDT in non-randomized studies

Study	Intervention, n/N (%)					Control, n/N (%)					Difference from Day 0 or difference-in-differences, in percentage points*			
	Day 0	Day 30	Day 60	Day 90	Day 180	Day 0	Day 30	Day 60	Day 90	Day 180	Day 30	Day 60	Day 90	Day 180
Fontoura 2016	59/821 (7.2%)	63/788 (8.0%)	46/799 (0.3%)	NA NA	44/832 (5.3%)	35/631 (5.6%)	35/626 (5.6)	2/634 (0.3%)	NA NA	9/676 (1.3%)	0.8%	3.8%	NA	2.3%
Searle 2020	88/2,215 (3.7%)	44/1,556 (2.8%)	NA	22/1,333 (1.7%)	NA	NA	NA	NA	NA	NA	-0.9%	NA	-2.1%	NA

* Estimates indicate the difference-in-differences for Fontoura 2016: [(%Pos-Intervention$_{post}$ – %Pos-Intervention$_{Day0}$) - (%Pos-Control$_{post}$ – %Pos-Control$_{Day0}$)] and differences from Day 0 (%Pos-Intervention$_{post}$ – %Pos-Intervention$_{Day0}$) for Searle 2020.

Table S5: Studies included for RACDT contextual factors data abstraction

Country Region	Title	Lead author Year	Accept-ability	Costs	Feasibility
Ethiopia, Senegal, Zambia (Modeling study)	Costing malaria interventions from pilots to elimination programmes	Galactionova 2020	0	1	0
Asia Pacific	Piloting a programme tool to evaluate malaria case investigation and reactive case detection activities: results from 3 settings in the Asia Pacific	Cotter 2017	0	1	1
Asia Pacific	Active case detection for malaria elimination: a survey among Asia Pacific countries	Smith-Gueye 2013	0	0	1
Bhutan	Development and evaluation of a spatial decision support system for malaria elimination in Bhutan	Wangdi 2016	0	0	1
Botswana	Malaria elimination in Botswana, 2012-2014: achievements and challenges	Chihanga 2016	0	0	1
Cambodia	Reactive case-detection of malaria in Pailin Province, Western Cambodia: lessons from a year-long evaluation in a pre-elimination setting	Hustedt 2016	0	0	1
Cambodia	Malaria elimination using the 1-3-7 approach: lessons from Sampov Loun, Cambodia	Kheang 2020	0	0	1
Cameroon	Adding proactive and reactive case detection into the integrated community case management system to optimise diagnosis and treatment of malaria in a high transmission setting of Cameroon: an observational quality improvement study	Bekolo 2019	1	0	0
China	Challenges in and lessons learned during the implementation of the 1-3-7 malaria surveillance and response strategy in China: a qualitative study	Lu 2016	0	0	1
China-Myanmar	Adapting the local response for malaria elimination through evaluation of the 1-3-7 system performance in the China-Myanmar border region	Wang 2017	0	0	1
Eswatini	Active Case Finding for Malaria: A 3-Year National Evaluation of Optimal Approaches to Detect Infections and Hotspots Through Reactive Case Detection in the Low-transmission Setting of Eswatini	Hsiang 2020	0	0	1
Ethiopia	Malaria case investigation with reactive focal testing and treatment: operational feasibility and lessons learned from low and moderate transmission areas in Amhara Region, Ethiopia	Bansil 2018	0	0	1
India	What is the value of reactive case detection in malaria control? A case-study in India and a systematic review	vanEijk 2016	0	0	1
Indonesia	Malaria risk factor assessment using active and passive surveillance data from Aceh Besar, Indonesia, a low endemic, malaria elimination	Herdiana 2016	0	1	0

Country Region	Title	Lead author Year	Accept-ability	Costs	Feasibility
	setting with Plasmodium knowlesi, Plasmodium vivax, and Plasmodium falciparum				
Indonesia	Costs and cost-effectiveness of malaria reactive case detection using loop-mediated isothermal amplification compared to microscopy in the low transmission setting of Aceh Province, Indonesia	Zelman 2018	0	1	0
Namibia	Effectiveness of reactive focal mass drug administration and reactive focal vector control to reduce malaria transmission in the low malaria-endemic setting of Namibia: a cluster-randomised controlled, open-label, two-by-two factorial design trial	Hsiang 2020	0	0	1
Namibia	Community acceptance of reactive focal mass drug administration and reactive focal vector control using indoor residual spraying, a mixed-methods study in Zambezi region, Namibia	Roberts 2021	1	0	0
Senegal	Scaling up malaria intervention "packages" in Senegal: using cost effectiveness data for improving allocative efficiency and programmatic decision-making	Faye 2018	0	1	0
Senegal	Mass testing and treatment for malaria followed by weekly fever screening, testing and treatment in Northern Senegal: feasibility, cost and impact	Conner 2020	0	1	0
Senegal	Case investigation and reactive case detection for malaria elimination in northern Senegal	Littrell 2013	1	0	0
Thailand	Active case detection with pooled real-time PCR to eliminate malaria in Trat province Thailand	Roawski 2012	0	1	0
Zambia	Evaluation of the operational challenges in implementing reactive screen-and-treat and implications of reactive case detection strategies for malaria elimination in a region of low transmission in southern Zambia	Searle 2016	0	0	1
Zambia	A qualitative review of implementer perceptions of the national community-level malaria surveillance system in Southern Province, Zambia	Lohfeld 2016	1	0	1
Zambia	A framework for evaluating the costs of malaria elimination interventions: an application to reactive case detection in Southern Province of Zambia, 2014	Larson 2016	0	1	0
Zambia	Malaria surveillance in low-transmission areas of Zambia using reactive case detection	Larsen 2015	0	1	0
Zambia	Improving the efficiency of reactive case detection for malaria elimination in southern Zambia: a cross-sectional study	Bhondoekhan 2020	0	0	1
Zanzibar	Malaria infection prevalence and sensitivity of reactive case detection in Zanzibar	Stuck 2020	0	0	1
Zanzibar	Operational coverage and timeliness of reactive case detection for malaria elimination in Zanzibar, Tanzania	VanDerHorst 2020	0	0	1
**Total**			**4**	**9**	**17**

Table S6: Studies included for RDA contextual factor data abstraction

Country	Title	Lead Author Year	Acceptability	Feasibility	Cost
**The Gambia**	Reactive, self-administered malaria treatment against asymptomatic malaria infection: results of a cluster randomized trial in The **Gambia**	Okebe, 2021	**Y**	**Y**	**N**
**Namibia**	Effectiveness of reactive focal mass drug administration and reactive focal vector control to reduce malaria transmission in the low malaria-endemic setting of **Namibia**: a cluster-randomised controlled, open-label, two-by-two factorial design trial	Hsiang 2020	**Y**	**Y**	**N**
**Eswatini**	Effectiveness and safety of reactive focal mass drug administration (rfMDA) using dihydroartemisinin-piperaquine to reduce malaria transmission in very low-endemic setting of **Eswatini**: a pragmatic cluster randomised controlled trial	Vilakati 2021	**N**	**Y**	**N**
**The Gambia**	Community perspectives on treating asymptomatic infections for malaria elimination in **The Gambia**	Jaiteh 2019	**Y**	**N**	**N**
**The Gambia**	Understanding adherence to reactive treatment of asymptomatic malaria infections in **The Gambia**	Jaiteh 2021	**N**	**Y**	**N**
**Namibia**	Community acceptance of reactive focal mass drug administration and reactive focal vector control using indoor residual spraying, a mixed-methods study in Zambezi region, **Namibia**	Roberts 2021	**Y**	**N**	**N**
**Zambia**	Treatment coverage estimation for mass drug administration for malaria with dihydroartemisinin-piperaquine in Southern Province, Zambia.	Finn 2020	**N**	**Y**	**N**
**Zambia**	Cost-effectiveness of focal mass drug administration and mass drug administration with dihydroartemisinin-piperaquine for malaria prevention in Southern Province, Zambia: results of a community-randomized controlled trial.	Yukich 2020	**N**	**N**	**Y**
**Zambia**	Adherence to Mass Drug Administration with Dihydroartemisinin-Piperaquine and Plasmodium falciparum Clearance in Southern Province, Zambia	Finn 2020	**N**	**Y**	**N**
**Zambia**	Assessment of the Acceptability of Testing and Treatment during a Mass Drug Administration Trial for Malaria in	Silumbe 2020	**Y**	**N**	**N**

	Zambia Using Mixed Methods				
**Eswatini**	"We were afraid of the lion that has roared next to us"; community response to reactive focal mass drug administration for malaria in **Eswatini** (formerly Swaziland)	Baltzell 2019	**Y**	**N**	**N**
**Total**			**6**	**6**	**1**

Table S7: Cost per person screened during RACDT

Country	General costs (without diagnostics)	Additional diagnostic costs			
		RDT	PCR	Microscopy	LAMP
**Indonesia(37)**	$11.00	Not reported	Not reported	$0.62	$16.00
**Senegal(53)**	$13.94	$0.36	Not reported	Not reported	Not reported
**Thailand(54)**	$3.96	Not reported	$1.25	Not reported	Not reported

Table S8: RACDT cost components from Indonesia and Senegal

Cost components	Indonesia (Aceh Province)(37)	Senegal (Richard Toll district)(38)
**% personnel**	41%	37%
**% training**	20%	29%
**% capital** (e.g., tablets, lab, vehicles, etc.)	13%	10%
**% consumables** (e.g., lab supplies, reagents, treatment)	9%	3%
**% other** (utilities, internet, communication, vehicle rental/fuel)	17%	N/A

Table S9: Community members' acceptance to participate in RACDT, in three countries in sub-Saharan countries

Country	Barriers/Challenges to acceptance	Solutions/reasons for success	Refusal rate
Community acceptance			
Namibia(25)	• Community members hesitation/resistance to RACDT during pre-trial interviews.<br>• A few concerns (superstitions) about blood draws	• Community engagement and sensitization appears to have helped participation"<br>• The study team's professionalism, and the respect shown for participants and local traditions were reported as critical for successful RACDT implementation	Year 1: 0.34% (3/894)<br><br>Year 2: 0.21% (10/4711)
Senegal (26) (Richard Toll district)		High participation was facilitated by:<br>• Advanced cascade sensitization whereby the health facility contacted the village health committee and requested that a committee member notify affected compounds on the evening prior to the investigation team visit<br>• Initial compound visits and booking appointments for follow-up with absent members; and<br>• return visits to the compound the same or next day.	2%
Zambia(27)	• Lack of community confidence in CHWs' ability to address diseases other than malaria<br>• Lack of community willingness to visit CHWs for malaria testing	• Provide notifications to alert HH members when RACDT would occur	

Table S10: Barriers, challenges, and solutions along the three steps of RACDT

	Barriers/Challenges	Solutions
**Index case detection and notification**		
Private Health Sector	• RACDT may not include passively detected cases at private health facilities (36)	• Engaging the private sector in malaria surveillance systems is critical, particularly in areas where many patients resort to private HFs, drug shops, or pharmacies (47)<br>• Collaborations with private providers is critical (28)
Public health sector		
Patient care seeking	• Delayed presentation of malaria patients to clinics(31)	
Inadequate preparation	• Village clinics not part of malaria web-based reporting system(31)	
Human Resources	• Limited diagnostic skills and shortage of primary health care staff (31)	• Need continuous capacity building (31)
Diagnostics	• Preference for RDTs (over microscopy) but they are not always available(31)<br>• Low sensitivity of RDTs(35)	
**Case investigation**		
Complexity of procedures	• Lack of standard operating procedures (SOPs)(31)	
Diagnostics	• Old microscopes, limited microscopy experience(31) Insufficient RDTs during high malaria season(48)	
Data completeness	• Difficulty classifying imported v. indigenous cases due to incomplete travel histories(31)	
Human resources	• During high season, a lower proportion of cases were followed up because CHWs were overwhelmed(48)	• During peak transmission times, the programme would benefit from additional CHWs or suspension of RACDT(4)
**Reactive case detection**		
Difficult to reach community and	• Timely follow up difficult in mountainous terrain or border areas with highly mobile populations (32)	

	Barriers/Challenges	Solutions
terrain	• Inaccessibility due to flooding(27)	• Provide CHWs with rain gear and boats(27)
Data completeness	• Incomplete case investigation forms limits follow-up(34)	
Cross-border issues (national and international)	• RACDT area extends beyond international border (49)<br>• Most cases are imported from outside the district, district-level response activities alone are likely to be ineffective in interrupting transmission(28)	• Strengthened cross-border collaborations needed to ensure adequate coverage of migrant and mobile populations(28)<br>• Communication and surveillance linkages with other operational district malaria response teams are necessary (28)
Diagnostics	• RDTs only detect Pf and miss other species and low-density infections(34, 35)<br>• Challenging to ensure a high quality of slides prepared by health centre staff (50)<br>• RDT stockouts prevented testing around index cases(23)	• Consider LAMP/more sensitive diagnostics(31)
Human Resources	• Lack of health facility workers to conduct malaria activities(36)<br>• At district level, lack of surveillance officers, resulting in inadequate supervision, case investigation and follow-up (34)<br>• Declining motivation among health workers to pursue case investigation and contact testing, particularly during weekends and public holidays (27, 28)<br>• Large numbers of households to screen (51) due to high density of people in small areas(33)	• Maintaining workforce motivation and need for consistent support, supervision and incentives(27)

Table S11: Proportion of household reached by RACDT

Country/Region	Proportion of households reached[2]	Proportion of households reached in a timely manner[3]
**China (Jiangsu)(36)**	19/19 (100%)	19/19 (100%)
**Indonesia (Ache)(36)**	57/58 (98%)	47/58 (81)
**Thailand (Ranong)(36)**	271/419 (65%)	229/271 (85%)
**Ethiopia (Amhara)(52)**	220/407 (54%) index cases identified, were investigated	N/A
**Zambia(52) (Kalomo, Choma and Namwala Districts)**	62%	N/A
**Zanzibar(47)**	49% of index case households	about 20% (inferred from Fig2)

Table S12: Adherence to RDA

	RDA coverage	Full adherence	Drug regimen	Adverse events
**Eswatini[1]**	62.4%	99.3% (n = 1099)	DHAp 3 days	Mild
**The Gambia[2]**	96.6%	98.5% (964/979)	DHAp 3 days	Mild to moderate
**The Gambia[3]**	N/A	91.6% (208/227)*	DHAp 3 days	N/A
**Zambia[4]**	N/A	92.8% overall (32,669/35,190) across four rounds, ranging from 91.5% to 95.6%	DHAp 3 days	5% stopped treatment early due to unspecified side effects (data for both MDA and RDA)

* According to self-report; adherence as defined by examining empty medicine bags and pills was 85.3% (233/273).

[2] Numerator is the number of RACDT events required based on local stratification criteria determining receptive areas.
[3] For China, Indonesia, and Thailand, target timeliness was within 7 days.

Supplemental figures

Figure S1: Risk of bias summary for randomized studies: review authors' judgements about each risk of bias item for each included study (RACDT and RDA)

	Randomization process	Recruitment of participants into clusters	Deviations from intended interventions	Missing outcome data: Parasitemia incidence	Missing outcome data: Parasitemia prevalence	Missing outcome data: Confirmed malaria incidence	Missing outcome data: Adverse events	Measurement of outcome: Parasitemia incidence	Measurement of outcome: Parasitemia prevalence	Measurement of outcome: Confirmed malaria incidence	Measurement of outcome: Adverse events	Selection of reported result: Parastiemia incidence	Selection of reported result: Parasitemia prevalence	Selection of reported result: Confirmed malaria incidence	Selection of reported result: Adverse events
Bridges 2021	+	?	+			+	−			+	−	+		?	+
Eisele 2020-HIGH	+	+	+	+	+	+		+	+	+		+	+	+	
Eisele 2020-LOW	+	+	+	+	+	+		+	+	+		+	+	+	
Hsiang 2020	+	+	+		+	+	?		+	+	−		+	+	+
Okebe 2021	+	+	+		+	+	−		+	?	−		+	+	+
Vilakati 2021	?	+	?			+	−			+	−			+	+

Note: 'Unclear risk of bias' should be interpreted as 'Some concerns'.

Figure S2: Risk of bias graph for RACDT randomized studies: review authors' judgements about each risk of bias item presented as percentages across all included studies

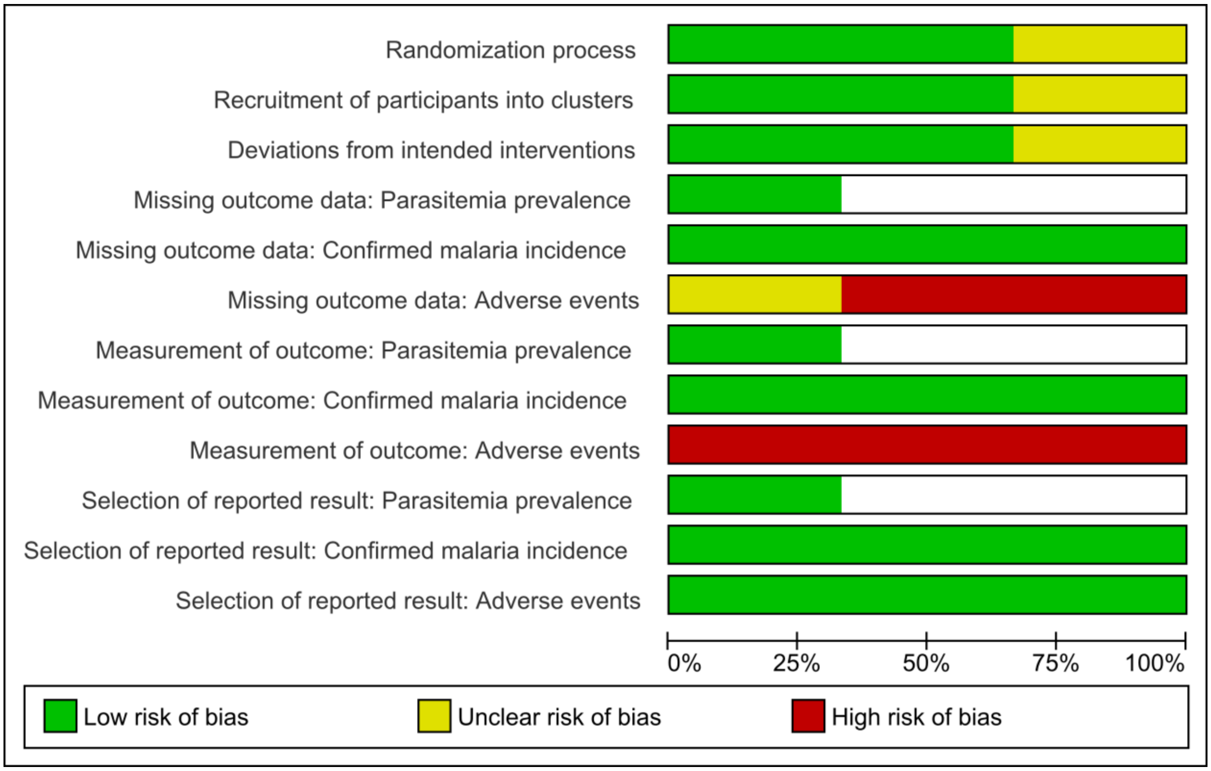

Note: 'Unclear risk of bias' should be interpreted as 'Some concerns'.

Figure S3: Risk of bias graph for RDA randomized studies: review authors' judgements about each risk of bias item presented as percentages across all included studies

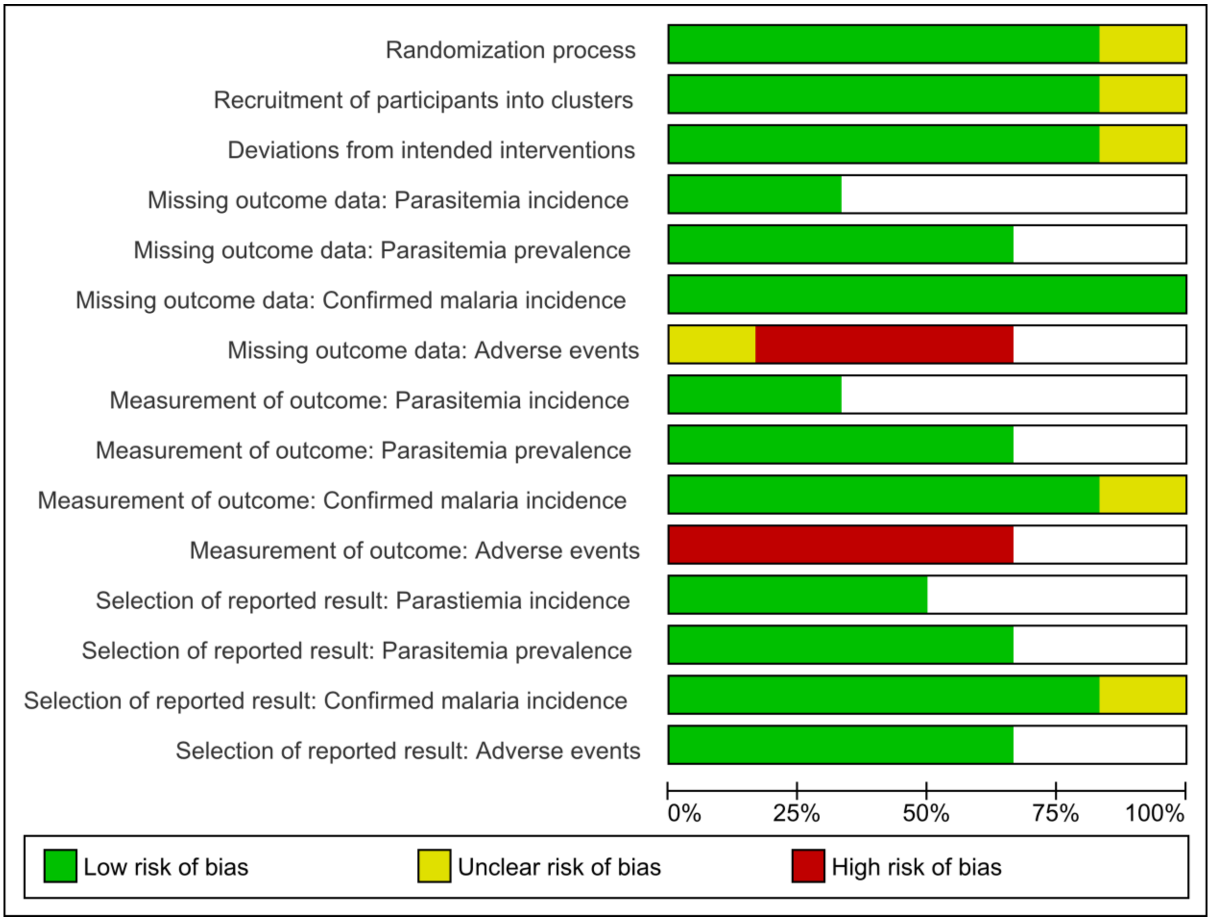

Note: ‘Unclear risk of bias’ should be interpreted as ‘Some concerns’.

Figure S4: Risk of bias summary for non-randomized RACDT studies: review authors' judgements about each risk of bias item for included study

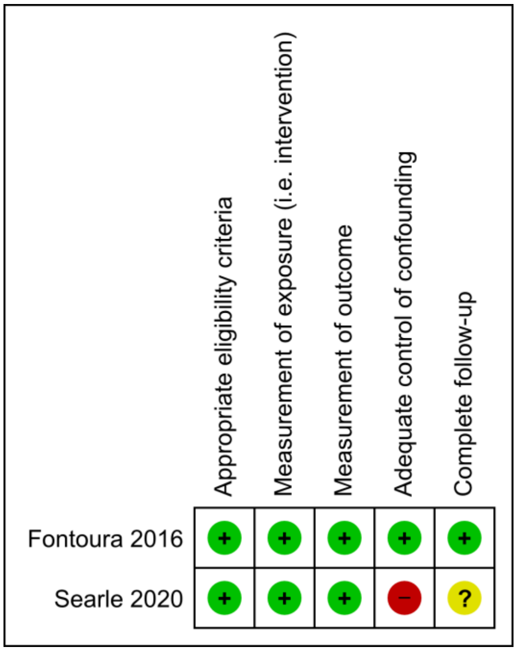

Note: ‘Unclear risk of bias’ should be interpreted as ‘Some concerns’.

Figure S5: Risk of bias graph for non-randomized RACDT studies: review authors' judgements about each risk of bias item presented as percentages across all included studies

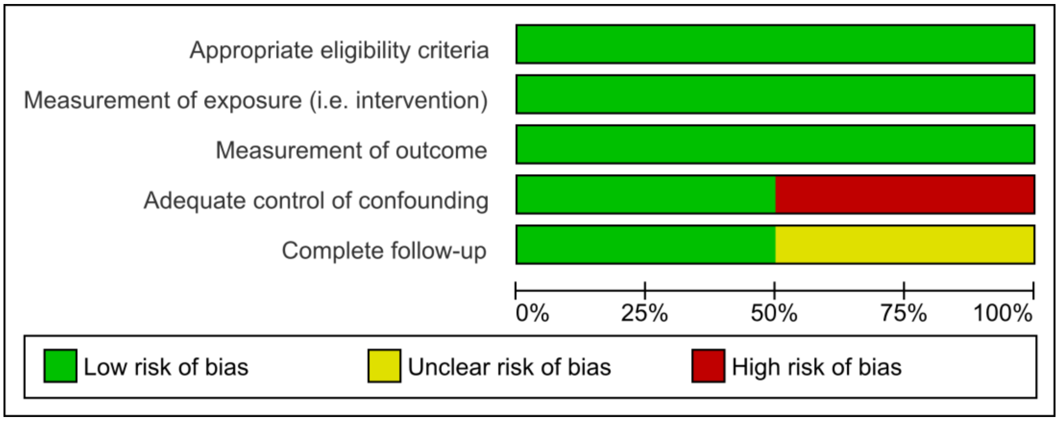

Note: ‘Unclear risk of bias’ should be interpreted as ‘Some concerns’.

Figure S6: Risk of bias summary for non-randomized RDA study: review authors' judgements about each risk of bias item for included study

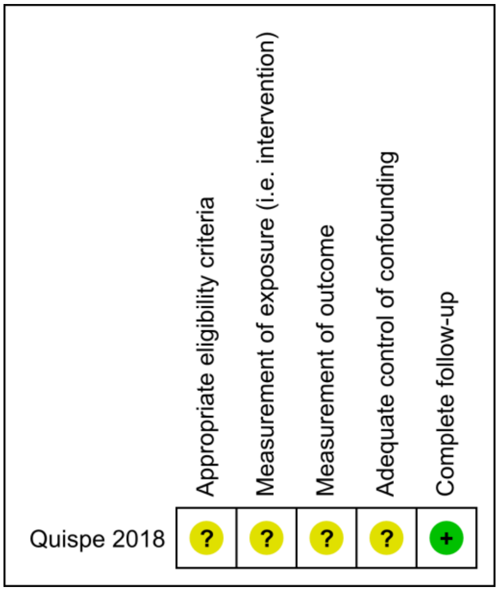

Note: 'Unclear risk of bias' should be interpreted as 'Some concerns'.

Figure S7: Risk of bias graph for non-randomized RDA study: review authors' judgements about each risk of bias item presented as percentages across all included studies

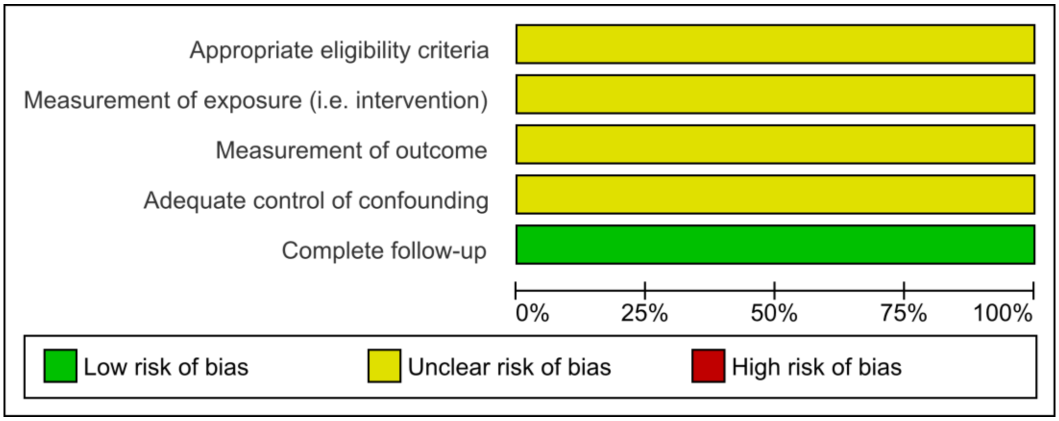

Note: 'Unclear risk of bias' should be interpreted as 'Some concerns'.

Figure S8: Forest plot of comparison: RDA versus no RDA/RACDT on prevalence of malaria infection omitting Eisele studies

Study or Subgroup	log[Odds Ratio]	SE	RDA Total	RACDT or Control Total	Weight	Odds Ratio IV, Fixed, 95% CI
Eisele 2020-HIGH (1)	-0.0834	0.425	698	770	0.0%	0.92 [0.40, 2.12]
Eisele 2020-LOW (2)	0.3075	0.4452	754	726	0.0%	1.36 [0.57, 3.25]
Hsiang 2020 (3)	-0.6162	0.3344	1863	2063	68.0%	0.54 [0.28, 1.04]
Okebe 2021 (4)	-0.3425	0.487	1452	1496	32.0%	0.71 [0.27, 1.84]
**Total (95% CI)**			**3315**	**3559**	**100.0%**	**0.59 [0.34, 1.01]**

Heterogeneity: Chi² = 0.21, df = 1 (P = 0.64); I² = 0%
Test for overall effect: Z = 1.92 (P = 0.06)

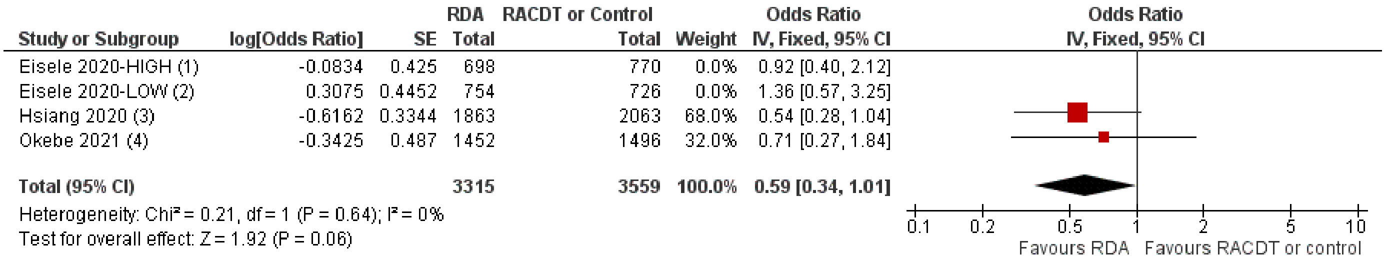

Footnotes

(3) The 95% CI lower limit is higher here than in the published paper (odds ratio = 0.54, 95% CI: **0.05**, 1.04), since the authors of the Namibia trial calculated the effect size using marginal effects post-estimation (to account for reactive IRS in half the clusters) after a regression model, and Review Manager software can only accommodate balanced CIs. Effect size from (non-linear) marginal effect post-estimation from generalized estimating equations (GEE) model using a logit function with variables for RDA, reactive IRS, the interaction between reactive IRS and RDA, and adjusted for 2016 incidence of local cases. Unadjusted effect size (from post-estimation marginal effect of RDA from GEE model using a logit function with variables for RDA, reactive IRS, the interaction between reactive IRS and RDA but no other covariates): 1.05 (0.03, 2.07).

(4) Random effects logistic regression (random effect for health facility) adjusted for age. Unadjusted odds ratio: 0.73 (95% CI: 0.27, 1.94).

Figure S9: Forest plot of comparison: RDA versus no RDA/RACDT on clinical malaria incidence omitting Eisele studies

Study or Subgroup	log[Rate Ratio]	SE	RDA Total	RACDT or Control Total	Weight	Rate Ratio IV, Fixed, 95% CI
Bridges 2021 (1)	-0.2231	0.2477	63000	56000	21.3%	0.80 [0.49, 1.30]
Eisele 2020-HIGH (2)	0.0296	0.1063	517895	779100	0.0%	1.03 [0.84, 1.27]
Eisele 2020-LOW (3)	-0.0408	0.1125	1376590	1402425	0.0%	0.96 [0.77, 1.20]
Hsiang 2020 (4)	-0.3425	0.2678	6255	7026	18.2%	0.71 [0.42, 1.20]
Okebe 2021 (5)	-0.2107	0.1594	8645	10330	51.3%	0.81 [0.59, 1.11]
Vilakati 2021 (6)	-0.1985	0.3758	44847	38018	9.2%	0.82 [0.39, 1.71]
**Total (95% CI)**			**122747**	**111374**	**100.0%**	**0.79 [0.63, 0.99]**

Heterogeneity: Chi² = 0.20, df = 3 (P = 0.98); I² = 0%
Test for overall effect: Z = 2.07 (P = 0.04)

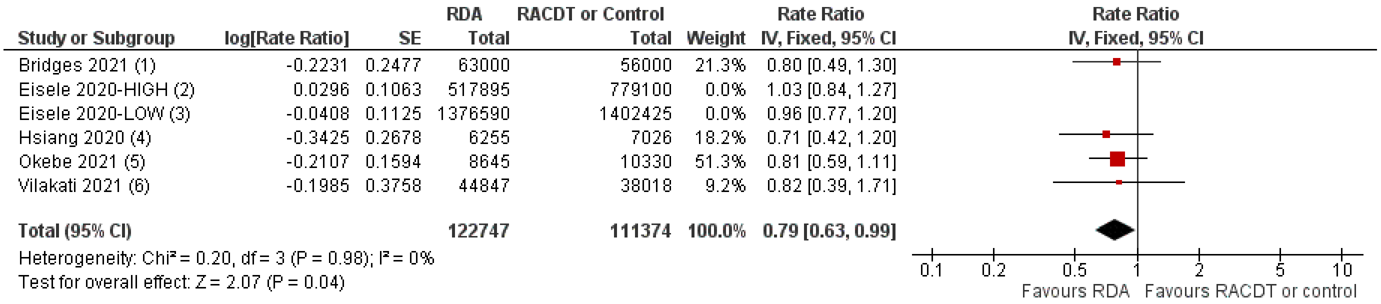

Footnotes

(1) Negative binomial analysis of monthly facility cases (random intercept for facility); adjusted for previous month's cases, normalized difference vegetation index (NDVI), precipitation, altitude, night-time light, number RDTs done each month, and seasonality (fourier term). Unadjusted estimate: 1.08 (95% CI: 0.78, 1.49).

(2) and (3) NA

(4) The 95% CI lower limit is higher here than in the published paper (rate ratio=0.71 (95% CI: **0.22**, 1.20). Effect size from (non-linear) marginal effect post-estimation from a negative binomial model with offset for cluster-level person time; variables for RDA, reactive vector control, interaction between RDA and reactive vector control, and adjusted for 2016 incidence of local cases. Unadjusted marginal effects from post-estimation (from unadjusted negative binomial model with terms for RACDT, reactive IRS, and the interaction between the two, with offset for cluster-level person time): 0.82 (0.26, 1.37).

(5) Poisson regression model adjusted for age. Unadjusted estimate from a logistic regression model (with a random effect for cluster): 1.04 (95% CI: 0.57, 1.91).

Figure S10: Forest plot of comparison: RDA versus no RDA/RACDT on confirmed malaria incidence for non-randomized studies

Study or Subgroup	log[Rate Ratio]	SE	RDA Total	Comparison Total	Weight	Rate Ratio IV, Fixed, 95% CI
Quispe 2018	-0.5334	0.1959	36231	163984	100.0%	0.59 [0.40, 0.86]
**Total (95% CI)**			**36231**	**163984**	**100.0%**	**0.59 [0.40, 0.86]**

Heterogeneity: Not applicable
Test for overall effect: Z = 2.72 (P = 0.006)

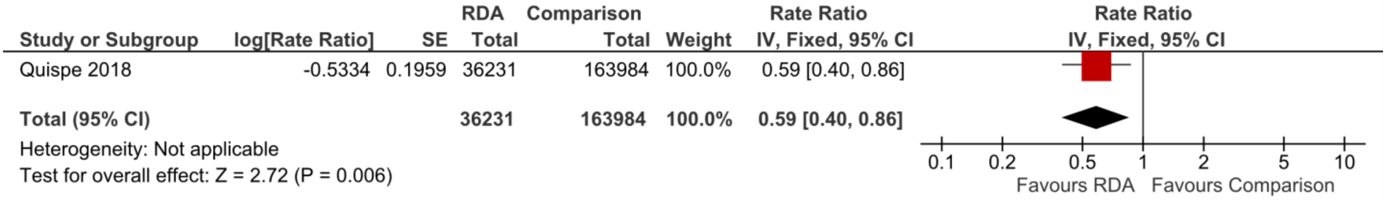

Footnotes

(1) Adjusted for seasonality and environmental factors including: soil moisture, surface pressure, and vegetation.
